# Sweet taste receptor agonists attenuate macrophage IL‐1β expression and eosinophilic inflammation linked to autophagy deficiency in myeloid cells

**DOI:** 10.1002/ctm2.1021

**Published:** 2022-08-21

**Authors:** Jinju Lee, So Jeong Kim, Go Eun Choi, Eunbi Yi, Hyo Jin Park, Woo Seon Choi, Yong Ju Jang, Hun Sik Kim

**Affiliations:** ^1^ Department of Biomedical Sciences Asan Medical Center University of Ulsan College of Medicine Seoul Korea; ^2^ Department of Clinical Laboratory Science Catholic University of Pusan Busan Korea; ^3^ Department of Otolaryngology Asan Medical Center University of Ulsan College of Medicine Seoul Korea; ^4^ Department of Microbiology Asan Medical Center University of Ulsan College of Medicine Seoul Korea; ^5^ Stem Cell Immunomodulation Research Center (SCIRC) Asan Medical Center University of Ulsan College of Medicine Seoul Korea

**Keywords:** autophagy deficiency, chronic rhinosinusitis, eosinophilic inflammation, IL‐1β, macrophage, sweet taste receptor

## Abstract

**Background:**

Eosinophilic inflammation is a hallmark of refractory chronic rhinosinusitis (CRS) and considered a major therapeutic target. Autophagy deficiency in myeloid cells plays a causal role in eosinophilic CRS (ECRS) via macrophage IL‐1β overproduction, thereby suggesting autophagy regulation as a potential therapeutic modality. Trehalose is a disaccharide sugar with known pro‐autophagy activity and effective in alleviating diverse inflammatory diseases. We sought to investigate the therapeutic potential of autophagy‐enhancing agent, trehalose, or related sugar compounds, and the underlying mechanism focusing on macrophage IL‐1β production in ECRS pathogenesis.

**Methods:**

We investigated the therapeutic effects of trehalose and saccharin on macrophage IL‐1β production and eosinophilia in the mouse model of ECRS with myeloid cell‐specific autophagy‐related gene 7 (*Atg7*) deletion. The mechanisms underlying their anti‐inflammatory effects were assessed using specific inhibitor, genetic knockdown or knockout, and overexpression of cognate receptors.

**Results:**

Unexpectedly, trehalose significantly attenuated eosinophilia and disease pathogenesis in ECRS mice caused by autophagy deficiency in myeloid cells. This autophagy‐independent effect was associated with reduced macrophage IL‐1β expression. Various sugars recapitulated the anti‐inflammatory effect of trehalose, and saccharin was particularly effective amongst other sugars. The mechanistic study revealed an involvement of sweet taste receptor (STR), especially T1R3, in alleviating macrophage IL‐1β production and eosinophilia in CRS, which was supported by genetic depletion of T1R3 or overexpression of T1R2/T1R3 in macrophages and treatment with the T1R3 antagonist gurmarin.

**Conclusion:**

Our results revealed a previously unappreciated anti‐inflammatory effect of STR agonists, particularly trehalose and saccharin, and may provide an alternative strategy to autophagy modulation in the ECRS treatment.

## Graphical abstract

Trehalose and saccharin attenuated eosinophilic inflammation linked to myeloid autophagy deficiency in a murine model of ECRS.

Activation of sweet taste receptor but not autophagy contributed to the alleviation of eosinophilia and IL‐1β dysregulation.

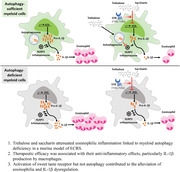

## 1 INTRODUCTION

Chronic rhinosinusitis (CRS) is a common upper airway disorder featured by sinonasal mucosal inflammation lasting for more than 12 weeks.^1^, ^2^ Due to its multifactorial etiology, CRS has a high degree of heterogeneity in inflammatory endotypes, clinical traits, and treatment outcomes.^2^, ^3^, ^4^ A range of inflammatory mediators and effector cells orchestrate in a complex manner, leading to the development of CRS with varying inflammatory endotypes and clinical phenotypes.^3^, ^4^, ^5^ Amongst the various inflammatory cells infiltrating the sinonasal mucosa of CRS patients, primary attention has been paid to the regulation of eosinophils due to their frequent association with refractory and recurrent CRS.^6^, ^7^ Accordingly, strategies that target eosinophil survival and migration in addition to topical or oral corticosteroids have been pursued as a promising treatment modality to resolve eosinophilic inflammation.^3^, ^8^, ^9^ However, the phenotypic and endotypic heterogeneity of CRS confound precise classification of the disease (e.g., often a mixed or alternative endotype instead of distinct T1 and T2 that is unrelated to polyp status) and thus challenge the predictions as to the best medical therapy being used for an individual patient.^4^, ^10^, ^11^, ^12^, ^13^

Autophagy is a conserved homeostatic mechanism that sequesters and clears damaged organelles and invaded pathogens via lysosomal degradation.^14^, ^15^ It also contributes to the cellular function of diverse immune cell types and its dysregulation is often linked to the progression of diverse inflammatory disorders.^16^, ^17^, ^18^, ^19^ Emerging evidence suggests that autophagy exerts a broad effect on Th cell polarisation and cytokine production, in part via control of innate immune cells in a context‐dependent manner.^17^ Autophagy deficiency in myeloid cells, especially macrophages, indirectly promoted the production of Th1 cytokine IFN‐γ in a model of GalN/LPS‐induced liver injury^20^ but also upregulate IL‐1β leading to enhanced generation of Th2 cytokines (IL‐4, IL‐5, IL‐13) in the inflamed tissues in eosinophilic CRS (ECRS) model.^21^ Autophagy deficiency in myeloid cells also enhanced Th17 responses in an active tuberculosis model.^22^ Given the capacity of autophagy to modulate diverse immune responses, it can be presumed autophagy modulation as a suitable therapeutic strategy to alleviate heterogeneous inflammatory disorders involving multiple endotypes such as CRS. However, few studies have been conducted to investigate the therapeutic potential of autophagy enhancer for the treatment of CRS, in particular ECRS.

In this study, using trehalose, a disaccharide with well‐documented pro‐autophagy activity in several cell types,^23^, ^24^, ^25^ we aimed to investigate the role of autophagy in the treatment of ECRS. We found that trehalose significantly ameliorates eosinophilia and disease pathogenesis in wild‐type (WT) ECRS mice but, unexpectedly, in myeloid cell‐specific autophagy‐deficient ECRS mice as well. Given that trehalose is a natural sugar, further study using different sweeteners revealed a significant therapeutic efficacy of both saccharin and trehalose in autophagy‐deficient ECRS mice through a mechanism involving macrophage IL‐1β inhibition and sweet taste receptor (STR) activation. Thus, our serendipitous finding uncovered a previously unappreciated anti‐inflammatory effect of STR agonists, particularly trehalose and saccharin, acting independently of autophagy, and suggests an alternative strategy for the treatment of ECRS.

## 2 RESULTS

### 2.1 Autophagy‐independent alleviation of eosinophilic CRS by trehalose

Our previous study found a causal relationship between autophagy dysfunction in myeloid cells, especially macrophages, and eosinophilia in CRS, suggesting the activation of autophagy as a promising therapeutic strategy to resolve eosinophilic sinonasal inflammation.^21^ Thus, we investigated whether autophagy‐inducing agent could alleviate eosinophilia and CRS pathogenesis. It is previously known that trehalose, a natural disaccharide with two glucose molecules, attenuates diverse inflammatory disorders by stimulating autophagy independently of the mammalian target of rapamycin (mTOR).^26^, ^27^, ^28^, ^29^, ^30^ Thus, we first tested the effect of trehalose on eosinophilic inflammation in autophagy‐intact mice in an established mouse model of ECRS.^21^, ^31^ Treatment with trehalose significantly ameliorated eosinophilic inflammation and anatomic abnormalities in the sinonasal tissue of mice with ECRS with respect to maximal mucosal thickness, epithelial hyperplasia, and the infiltration of eosinophils (Figure S1A,B). This therapeutic benefit of trehalose appeared more pronounced with local intranasal injection (100 mg/kg) compared to systemic intraperitoneal injection (2 g/kg). Consistent with our previous study,^21^ a noticeable increase in the staining of LC3B, a common autophagy marker, often in colocalisation with CD68+ macrophages in the CRS group was observed (Figure S1C,D). As expected, such LC3B staining was further enhanced by the treatment with trehalose, particularly via intranasal administration. Together, these results indicated a therapeutic effect of trehalose on eosinophilia and CRS pathogenesis in the mouse model.

**FIGURE 1:**
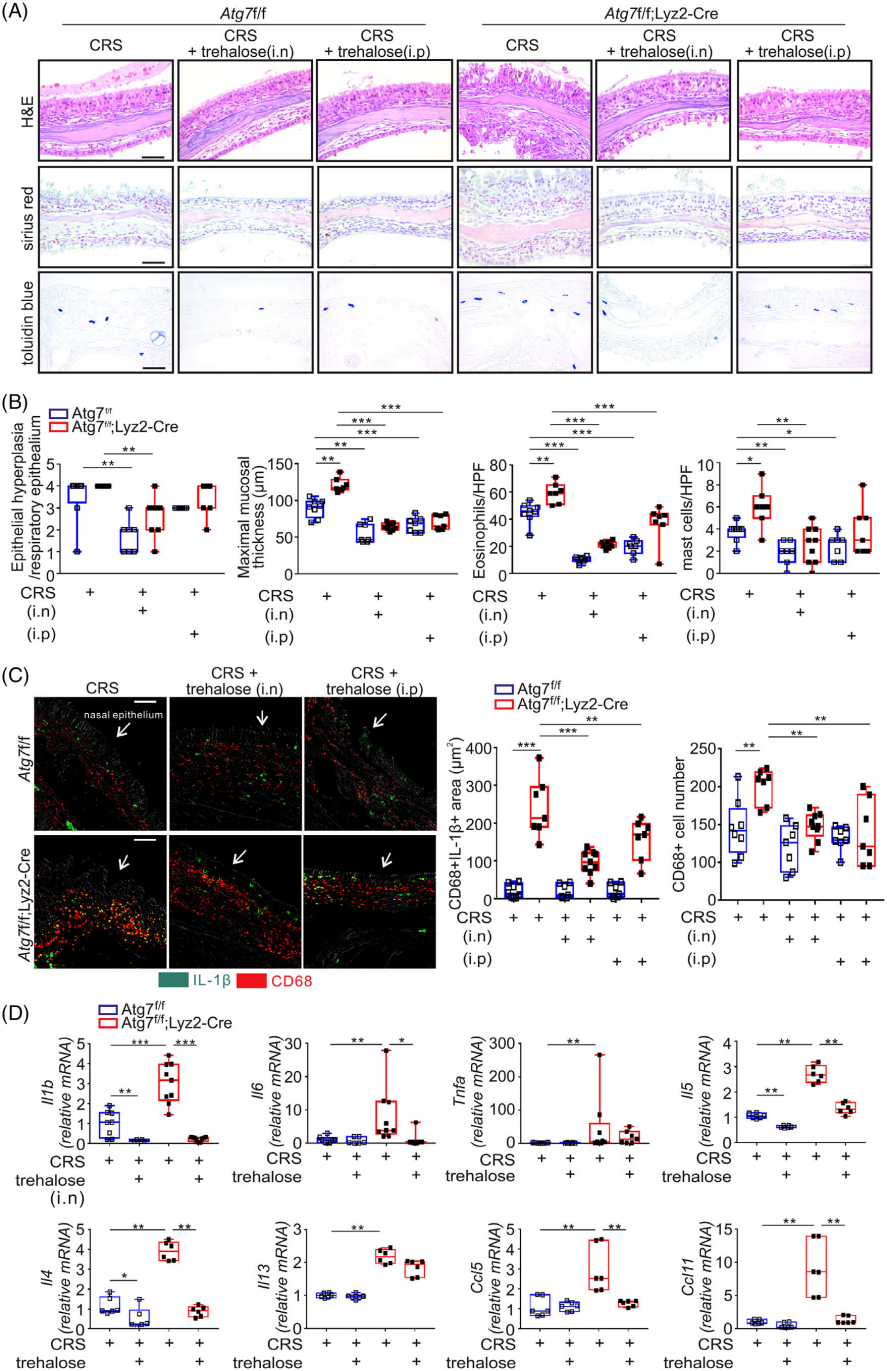
Trehalose induces autophagy‐independent alleviation of eosinophilic inflammation in chronic rhinosinusitis (CRS). (A) Effect of trehalose via intranasal (i.n.) or intraperitoneal (i.p.) injection in a murine model of ECRS. Representative schematics of hematoxylin and eosin (H & E; upper)‐, Sirius red (middle)‐, and acidic toluidine blue (lower)‐stained tissue sections. (B) Scores of epithelial hyperplasia, maximal mucosal thickness in H&E‐stained tissue sections, Sirius red‐positive eosinophil counts of the lamina propria in Sirius red‐stained tissue sections, and toluidine blue‐positive mast cell counts in acidic toluidine blue‐stained tissue sections. (C) Representative dual‐immunofluorescence staining for IL‐1β and CD68‐positive macrophages in sinonasal tissue from each group of mice (left). Yellow signals indicate colocalisation of two marker proteins. The statistical bar charts show the area of colocalisation (middle) and the number of CD68‐positive macrophages (right). Arrow indicates the nasal epithelium. (D) Relative mRNA levels corresponding to the indicated proteins were determined by using qRT‐PCR and normaliaed to β‐actin mRNA. Scale bars = 50 μm. Data are expressed as box‐and‐whisker plot with the box marking 25th, median, and 75th percentiles (*n* = 7–9 per group). *P < .05, **P < .01, and ***P < .005, One‐way ANOVA or Mann–Whitney *U*‐test

Next, we determined the involvement of autophagy in the therapeutic benefit of trehalose using mice harbouring a selective deletion of autophagy‐related gene (*Atg7*) in myeloid cells (hereinafter referred to as *Atg7* ^fl/fl^;*Lyz2*‐Cre mice). This mouse has a selective deficiency of the vital autophagy gene *Atg7* in cells of myeloid origin, such as neutrophils and macrophages but not eosinophils,^32^, ^33^ infiltrating the sinonasal tissue during the development of CRS. Autophagy‐deficiency in myeloid cells was confirmed by the negligible level of Atg7 protein in macrophages isolated from *Atg7* ^fl/fl^;*Lyz2*‐Cre mice (Figure S2A). Consistent with a previous report,^21^ we observed that autophagy deficiency in myeloid cells aggravates eosinophilia, infiltration of mast cells driving eosinophilic inflammation,^34^ and CRS severity, such as mucosal thickening (Figure 1A,B). Treatment with trehalose significantly alleviated the infiltration of eosinophils and mast cells as well as tissue abnormalities in autophagy‐intact *Atg7* ^fl/fl^ ECRS mice but, surprisingly, in autophagy‐deficient *Atg7* ^fl/fl^;*Lyz2*‐Cre ECRS mice as well (Figure 1A,B). Supporting this, we observed a marked increase in blood eosinophilia amongst leukocytes examined in *Atg7* ^fl/fl^;*Lyz2*‐Cre ECRS mice, which was diminished by the intranasal treatment with trehalose (Figure S2B). This indicated that trehalose has therapeutic potency in the alleviation of ECRS independently of macrophage and neutrophil autophagy, particularly when intranasally injected.

We previously observed that ECRS aggravation by autophagy deficiency is causally linked to elevated levels of IL‐1β from macrophages amongst other myeloid cells.^21^ We found that IL‐1β staining alone or colocalised with CD68+ macrophages was substantially augmented in *Atg7* ^fl/fl^;*Lyz2*‐Cre ECRS mice compared to *Atg7* ^fl/fl^ ECRS mice, which was significantly diminished following trehalose treatment regardless of *Atg7*‐deficiency (*P* < 0.001 for intranasal injection vs. *P* < 0.01 for intraperitoneal injection; Figure 1C, Figure S2C). Likewise, the trehalose treatment significantly reduced the infiltration of CD68+ macrophages in *Atg7* ^fl/fl^;*Lyz2*‐Cre ECRS mice (Figure 1C). Given more efficacious effect via local intranasal injection (100 mg/kg) rather than intraperitoneal injection (2 g/kg), intranasal injection was chosen for further experiments in view of safety and effective dose. Supporting anti‐inflammatory effect of trehalose, we detected a significant increase in mRNA levels of IL‐1β, IL‐6, TNF‐α by *Atg7*‐deficiency, as assessed by using qRT‐PCR, amongst which IL‐1β and IL‐6 were remarkably suppressed by the intranasal trehalose treatment (Figure 1D). The same treatment also significantly reduced the expression of genes linked to Th2 response and eosinophilia (*Il4* and *Il5 but not Il13*) as well as eosinophil recruitment (*Ccl5* and *Ccl11*) in *Atg7* ^fl/fl^;*Lyz2*‐Cre ECRS mice. In comparison, trehalose did not affect LPS‐mediated upregulation of CD69 on mouse blood eosinophils and mRNA levels of IL‐5 and IL‐13 in human eosinophilic cell line (Figure S3A,B), suggesting that trehalose may not directly act on eosinophils to alleviate eosinophilia. Given comparable efficacy between the trehalose treatment and macrophage IL‐1β blockade^21^ for reducing mast cell infiltration and eosinophilia, we speculate the suppressive effect of trehalose on macrophage IL‐1β as a major mechanism of action, although its potential effect on other cells producing type 2 cytokines cannot be excluded. Collectively, our results suggested a therapeutic efficacy of trehalose in alleviating eosinophilia and CRS via suppression of inflammatory responses entailing macrophage IL‐1β in an autophagy‐independent manner.

### 2.2 Autophagy‐independent suppression of inflammatory cytokine production by trehalose

**FIGURE 2:**
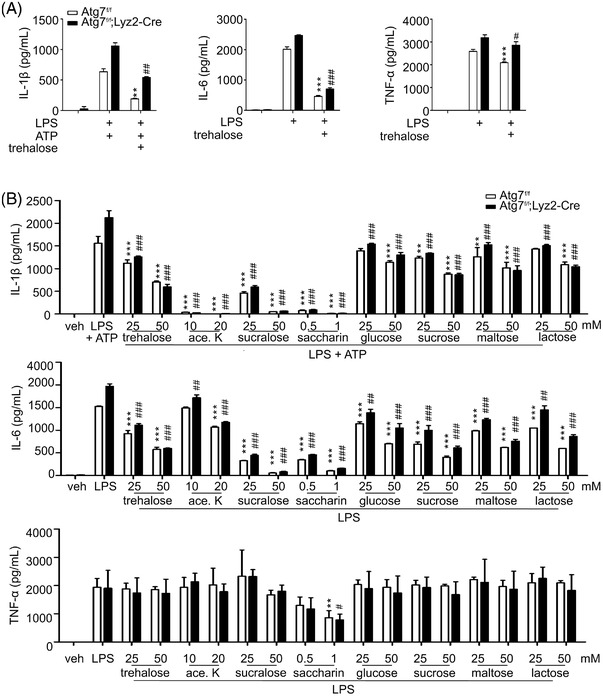
Trehalose and saccharin suppress inflammatory cytokine production from autophagy‐deficient macrophages. (A) Effect of trehalose on the levels of IL‐1β, IL‐6, and TNF‐α in culture supernatants of activated peritoneal macrophages from *Atg7^fl/fl^* or *Atg7^fl/fl^;Lyz2*‐Cre mice, as determined by ELISA. (B) Effect of the indicated sugars on the levels of IL‐1β (upper), IL‐6 (middle), and TNF‐α (bottom) in culture supernatants of activated peritoneal macrophages from *Atg7^fl/fl^* or *Atg7^fl/fl^;Lyz2*‐Cre mice, as determined by ELISA. Data are expressed as means ± SDs. **P < .01, ***P < .005, #*P* < .05, ##*P* < .01, and ###*P* < .005, Student's *t*‐test or one‐way ANOVA. Data are representative of at least three independent experiments performed in duplicate or triplicate

To investigate the underlying mechanism, we assessed the direct effect of trehalose on macrophage production of IL‐1β, IL‐6, and TNF‐α. Peritoneal macrophages (PM) were stimulated by Lipopolysaccharide (LPS) with or without trehalose pretreatment, and the production of IL‐1β, IL‐6, and TNF‐α was measured by means of Enzyme‐linked immunosorbent assay (ELISA). IL‐1β, but not IL‐6 and TNF‐α, production was significantly enhanced in autophagy‐deficient macrophages exposed to LPS, an observation compatible with previous studies.^35^, ^36^ Notably, the trehalose treatment (50 mM) significantly reduced the production of IL‐1β and IL‐6 and to a lesser extent TNF‐α from activated macrophages; the effect was comparable between WT and *Atg7*‐deficient macrophages (Figure 2A). To further characterise this autophagy‐independent response, we treated *Atg7*‐deficient macrophages with various doses of trehalose (25–100 mM). Both WT and *Atg7*‐deficient macrophages exposed to trehalose had significantly and dose‐dependently reduced levels of cytokines; the effect was most pronounced for IL‐1β but only apparent for TNF‐α at high concentration (100 mM) (Figure S4). Thus, these results indicated a direct effect of trehalose on the suppression of cytokine production, particularly IL‐1β, independently of autophagy.

### 2.3 Anti‐inflammatory potential of natural and artificial sugars in autophagy‐deficient macrophages

As trehalose is a natural sugar consisting of two molecules of glucose, we next assessed the effect of various natural sugars (glucose, sucrose, maltose and lactose) and artificial sweeteners (acesulfame‐K, sucralose and saccharin) on IL‐1β, IL‐6, and TNF‐α production. Of interest, all of the sugars tested reduced the generation of IL‐1β and IL‐6 rather than TNF‐α by activated macrophages from both *Atg7* ^fl/fl^ and *Atg7* ^fl/fl^;*Lyz2*‐Cre mice (Figure 2B). Compared to the modest effect of natural sugars, artificial sweeteners except acesulfame‐K markedly diminished IL‐1β and IL‐6 production from *Atg7*‐deficient macrophages; acesulfame‐K had a potent effect on IL‐1β, but not IL‐6, production. This anti‐inflammatory effect was most pronounced with saccharin even at low concentrations (0.5–1 mM). Thus, these results suggest a common anti‐inflammatory potential of diverse sugars by the inhibition of IL‐1β production, which was independent of autophagy. Given the most potent effect of saccharin, we focused our study on saccharin in addition to trehalose in the context of IL‐1β production. Using various doses of saccharin (25–500 μM), we observed a dose‐dependent and significant reduction of IL‐1β but, to a much lesser extent, IL‐6 and TNF‐α production from *Atg7*‐deficient macrophages (Figure S5). Trehalose and saccharin did not significantly influence macrophage viability or proliferation during the assay (data not shown), hence excluding their toxic effect on macrophages.

### 2.4 Inhibition of pro‐IL‐1β expression by trehalose and saccharin

**FIGURE 3:**
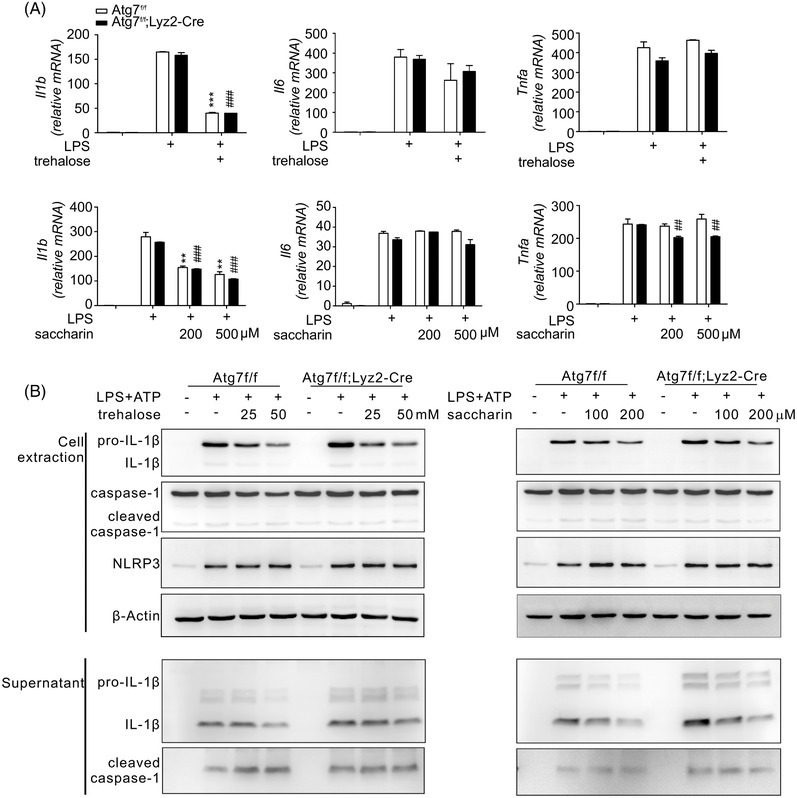
Trehalose and saccharin regulate the pro‐IL‐1β expression but not the NLRP3 inflammasome. (A) Effect of trehalose (top) or saccharin (bottom) on relative mRNA levels corresponding to IL‐1β, IL‐6, and TNF‐α was determined by using qRT‐PCR and normalised to β‐actin mRNA. (B) Effect of trehalose (top) or saccharin (bottom) on the relative protein levels of the NLRP3 inflammasome. Lysates and culture supernatants were prepared from activated peritoneal macrophages from *Atg7^fl/fl^* or *Atg7^fl/fl^;Lyz2*‐Cre mice and immunoblotted for the indicated proteins. Data are expressed as means ± SDs. **P < .01, ***P < .005, and ###*P* < .005, Student's *t*‐test or one‐way ANOVA. Data are representative of at least three independent experiments performed in duplicate or triplicate

To probe the mechanism underlying the suppression of cytokine production by trehalose and saccharin, we first assessed the expression of IL‐1β, IL‐6, TNF‐α gene in macrophages. Using qRT‐PCR, we found that both trehalose (50 mM) and saccharin (0.2–0.5 mM) significantly suppress mRNA expression of IL‐1β but marginally reduce that of IL‐6 and TNF‐α (Figure 3A). Thus, we focused our study on the autophagy‐independent regulation of IL‐1β by trehalose and saccharin, given the causal association of autophagy‐deficient macrophages overexpressing IL‐1β with exacerbation of ECRS.^21^ IL‐1β is synthesised in an inactive precursor form (pro‐IL‐1β) primarily by activated macrophages and monocytes, which needs to be cleaved into a biologically active form by caspase‐1 via recruitment to a multiprotein signalling complex termed the inflammasome (e.g., NLRP3 inflammasome).^37^ In general, inflammasome activation in murine macrophages occurs in the two‐step process such as priming and then activation.^37^ The priming step is triggered by various inflammatory stimuli (e.g., LPS) and serves to upregulate the expression of Nucleotide‐binding oligomerization domain, Leucine rich Repeat and Pyrin domain containing Proteins (NLRP3) and pro‐IL‐1β, and the activation step is triggered by NLRP3 activator including ATP, thus promoting the assembly of NLRP3 inflammasome and activated caspase 1‐induced IL‐1β production. In particular, NLRP3, caspase‐1, and the downstream IL‐1β are found to be overexpressed in ECRS with nasal polyps (ECRSwNP)^38^ and are required for allergen‐specific Th2 responses and eosinophilic lung inflammation.^39^ We found that trehalose and saccharin dose dependently suppress the levels of pro‐IL‐1β in cell extracts of WT and *Atg7*‐deficient macrophages, correlating with the production of mature IL‐1β in culture supernatant (Figure 3B). In comparison, treatment with trehalose and saccharin had no effect on protein levels of caspase‐1 and NLRP3 in cell extracts or active caspase‐1 cleaved form in culture supernatant (Figure 3B). Thus, these findings together suggest an inhibition of pro‐IL‐1β expression at the level of mRNA and protein as a potential mechanism in the autophagy‐independent suppression of IL‐1β by trehalose and saccharin.

### 2.5 Involvement of taste receptor type 1 member 3 in suppressing IL‐1β production

**FIGURE 4:**
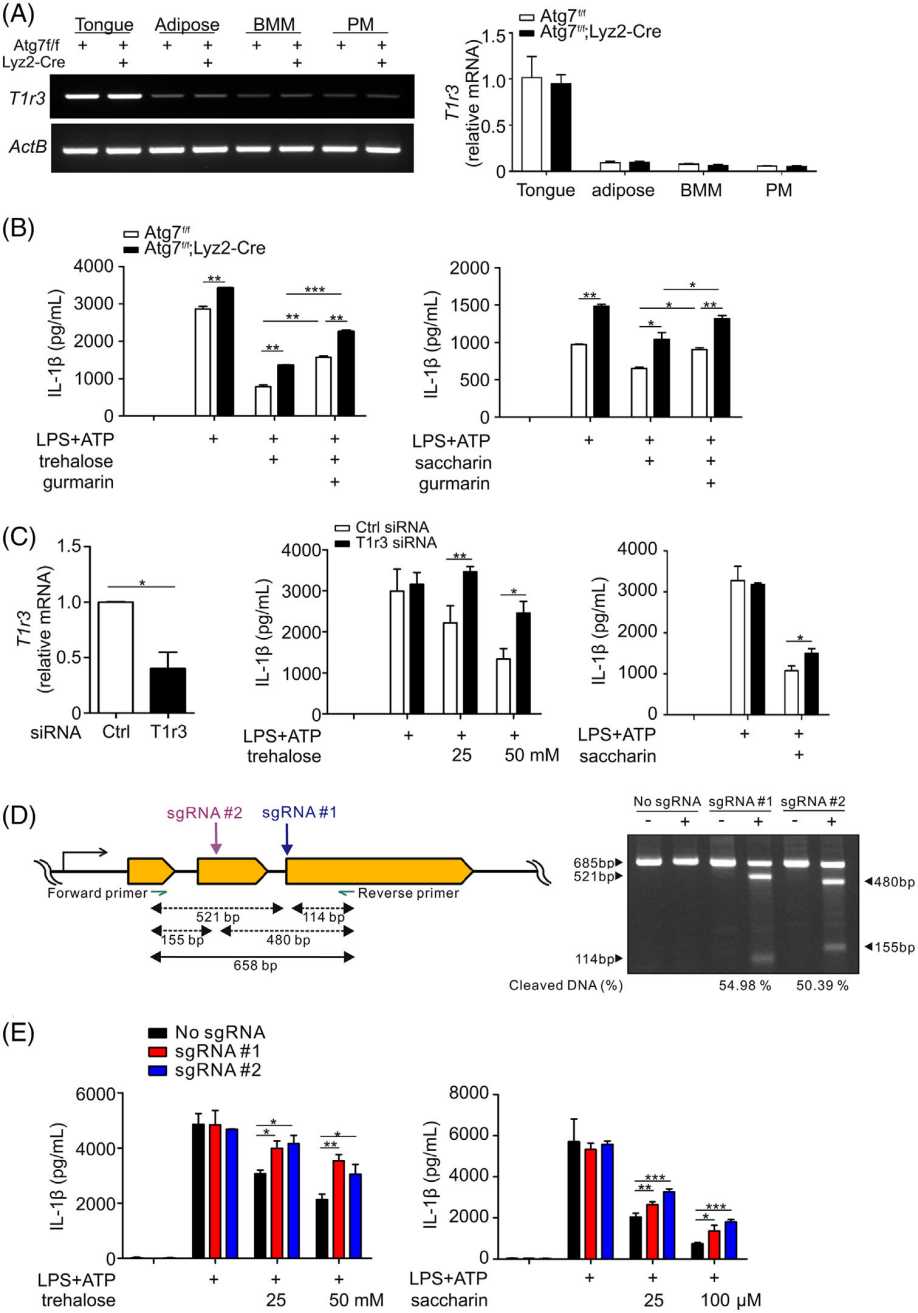
Taste receptor T1R3 contributes to the suppression of IL‐1β production. (A) Relative mRNA levels of T1R3 were determined by using PCR (left) or qRT‐PCR (right) with normalisation to β‐actin mRNA. (B) Effect of T1R3 blockade via treatment with gurmarin on trehalose (left)‐ or saccharin (right)‐mediated suppression of IL‐1β levels in culture supernatants of activated peritoneal macrophages from *Atg7^fl/fl^* or *Atg7^fl/fl^;Lyz2*‐Cre mice, as determined by ELISA. (C) siRNA‐mediated knockdown of T1R3 in *Atg7*‐deficient BMM was determined by using qRT‐PCR and normalised to β‐actin mRNA (left). Effect of T1R3 knockdown on trehalose (middle)‐ or saccharin (right)‐mediated suppression of IL‐1β levels in culture supernatants of activated BMM, as determined by ELISA. (D) Scheme of CRISPR/Cas9‐mediated knockout of T1R3 with specific sgRNA #1 or sgRNA #2 (left). Representative gel image showing T1R3 knockout efficiency in *Atg7*‐deficient BMM, as determined by T7E1 cleavage assay (right). (E) Effect of T1R3 knockout on trehalose (left)‐ or saccharin (right)‐mediated inhibition of IL‐1β levels in culture supernatants of activated *Atg7*‐deficient BMM, as determined by ELISA. Data are expressed as means ± SDs. *P < .05, **P < .01, and ***P < .005, Student's *t*‐test. Data are representative of at least three independent experiments performed in duplicate or triplicate

As trehalose and saccharin function as STR agonists, we investigated the possible involvement of STR in either trehalose or saccharin‐mediated suppression of IL‐1β expression. STR is a heterodimer of the G protein‐coupled receptor (GPCR) family comprised of taste receptor type 1 member 2 (T1R2) and T1R3, which responds to a broad range of sweet compounds including natural sugars and artificial sweeteners including saccharin and acesulfame‐K.^40^, ^41^ T1R3 also forms a heterodimer with T1R1 and functions as umami taste receptors for diverse L‐amino acids.^42^ A previous report demonstrated that T1R3 alone or in combination with T1R2 or T1R1 is sufficient to elicit a response to trehalose.^43^ Accordingly, to test T1R3 involvement, the effect of trehalose and saccharin was compared with that of L‐serine, which stimulates T1R1/T1R3, on IL‐1β production by autophagy‐deficient macrophages. Although less potent, L‐serine (100 mM) also recapitulated the selective effect of trehalose (50 mM) and saccharin (100 μM) on the production of IL‐1β but not TNF‐α (Figure S6). We observed the expression of T1R3 mRNA in both bone marrow‐derived macrophages (BMM) and PM (Figure 4A), compatible with the finding of a previous study.^44^ T1R3 expression in macrophages was far lower than that observed in the mouse tongue as a positive control but was comparable to that of adipose tissue, and was independent of autophagy deficiency (Figure 4A).

To assess the functional role of T1R3 in trehalose‐ or saccharin‐mediated effect, activated PMs were treated with trehalose or saccharin in the absence or the presence of gurmarin, a selective T1R3 antagonist in rodents.^45^, ^46^ Gurmarin partially but significantly reversed the effect of trehalose (50 mM) and saccharin (100 μM) on IL‐1β production (Figure 4B). Gurmarin per se did not influence IL‐1β production from and viability of activated PM (data not shown). On the basis of this promising result, we performed genetic depletion approaches using siRNA or the CRISPR/Cas9 system targeting T1R3 in BMM to directly investigate the role of T1R3 (Figure 4C–E). As a control experiment, we assessed whether trehalose and saccharin have a comparable effect on the regulation of IL‐1β and TNF‐α in BMM. As observed with PM, trehalose and saccharin preferentially reduced the production of IL‐1β rather than TNF‐α from *Atg7*‐deficient BMM (Figure S7). For siRNA‐mediated knockdown, BMM from *Atg7* ^fl/fl^;*Lyz2*‐Cre mice were transfected with T1R3‐specific siRNA, which led to a significant decrease in T1R3 mRNA after 72 h (Figure 4C). Notably, trehalose (25–50 mM) and saccharin (100 μM) suppressed the production of IL‐1β from *Atg7*‐deficient BMM, which was significantly restored by the T1R3 knockdown (Figure 4C).

To further determine the role of T1R3 in such a context, we conducted CRISPR‐Cas9‐mediated T1R3 gene knockout in *Atg7*‐deficient BMM. Two different single‐guide RNAs (sgRNA) complementary to exon 3 spanning 6 bp of intron (#1) and to exon 2 (#2) of the T1R3 gene were complexed with Cas9 to form RNPs, respectively, and were then introduced into *Atg7*‐deficient BMM (Figure 4D). We confirmed an introduction of the mutation at the target site of the T1R3 gene using an T7E1 cleavage assay, which revealed a 50%–55% DNA cleaved ratio with sgRNA #1 and sgRNA #2, respectively (Figure 4D). Despite the modest editing efficiencies, T1R3 gene knockout with both sgRNAs partially but significantly restored the IL‐1β production that was reduced by treatment with trehalose and saccharin (Figure 4E), consistent with the siRNA results. These results collectively suggested that trehalose and saccharin suppress the inflammatory responses of macrophages, particularly IL‐1β, through a pathway involving T1R3 but not autophagy.

### 2.6 Blocking T1R3 by gurmarin aggravates ECRS

**FIGURE 5:**
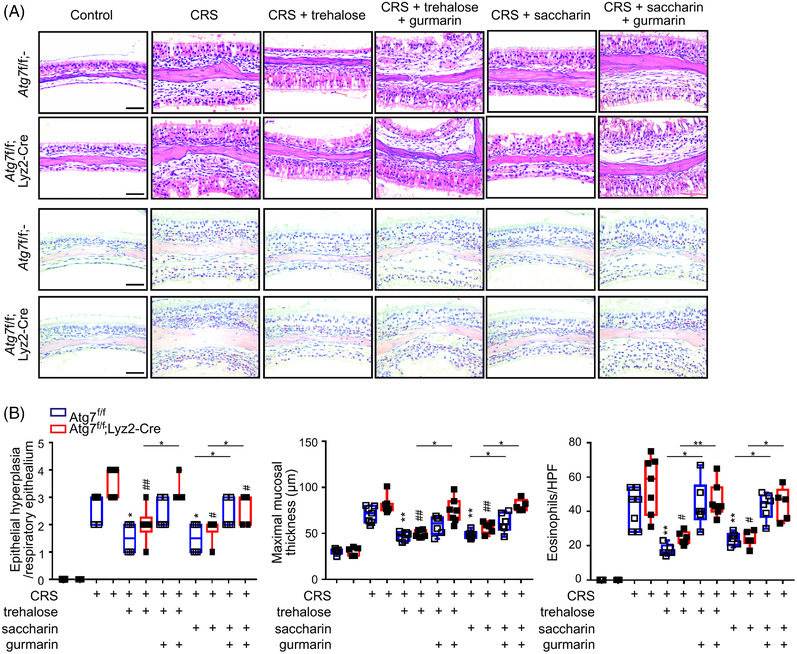
Blocking of T1R3 by gurmarin exacerbates eosinophilic CRS (ECRS). (A) Effect of T1R3 blockade via treatment with gurmarin on trehalose‐ or saccharin‐mediated alleviation of ECRS. Representative photographs of hematoxylin and eosin (H&E)‐ and Sirius red‐stained tissue sections. (B) Scores of epithelial hyperplasia, maximal mucosal thickness in H&E‐stained tissue sections, and eosinophil counts of the lamina propria in Sirius red‐stained tissue sections. Scale bars = 50 μm. Data are expressed as box‐and‐whisker plot with the box marking 25th, median, and 75th percentiles (*n* = 5–7 per group). *P < .05, **P < .01, #*P* < .05, and ##*P* < .01, Mann–Whitney *U*‐test

Next, we investigated whether T1R3 is involved in the therapeutic effect of trehalose and saccharin on ECRS with the T1R3 antagonist gurmarin. We observed no significant difference in mRNA expression of T1R3 receptor during the ECRS development (Figure S8). As a control experiment, we validated the therapeutic efficacy of saccharin in the ECRS treatment. We observed that the treatment with saccharin (2 mg/kg) effectively ameliorated the tissue abnormalities and eosinophil infiltration into the sinus mucosa of mice with ECRS (Figure S9). To assess the effect of T1R3 blocking in vivo, gurmarin was administered to *Atg7* ^fl/fl^;*Lyz2*‐Cre mice as well as *Atg7* ^fl/fl^ mice treated with trehalose or saccharin during the development of ECRS. Trehalose (100 mg/kg) and saccharin (2 mg/kg) significantly alleviated the maximal mucosal thickness and eosinophilia in the sinonasal mucosa of *Atg7* ^fl/fl^ and *Atg7* ^fl/fl^;*Lyz2*‐Cre ECRS mice, which were significantly exacerbated by gurmarin administration (Figure 5A,B). Furthermore, we found that gurmarin administration markedly ablated the effect of trehalose and saccharin on the reduction of IL‐1β‐producing CD68+ macrophages lacking autophagy in myeloid cells (Figure S10). Collectively, our results suggested an involvement of T1R3 in the alleviation of eosinophilic inflammation by trehalose or saccharin through an effect on macrophage IL‐1β production.

### 2.7 Involvement of human T1R3 in the suppression of IL‐1β production

**FIGURE 6:**
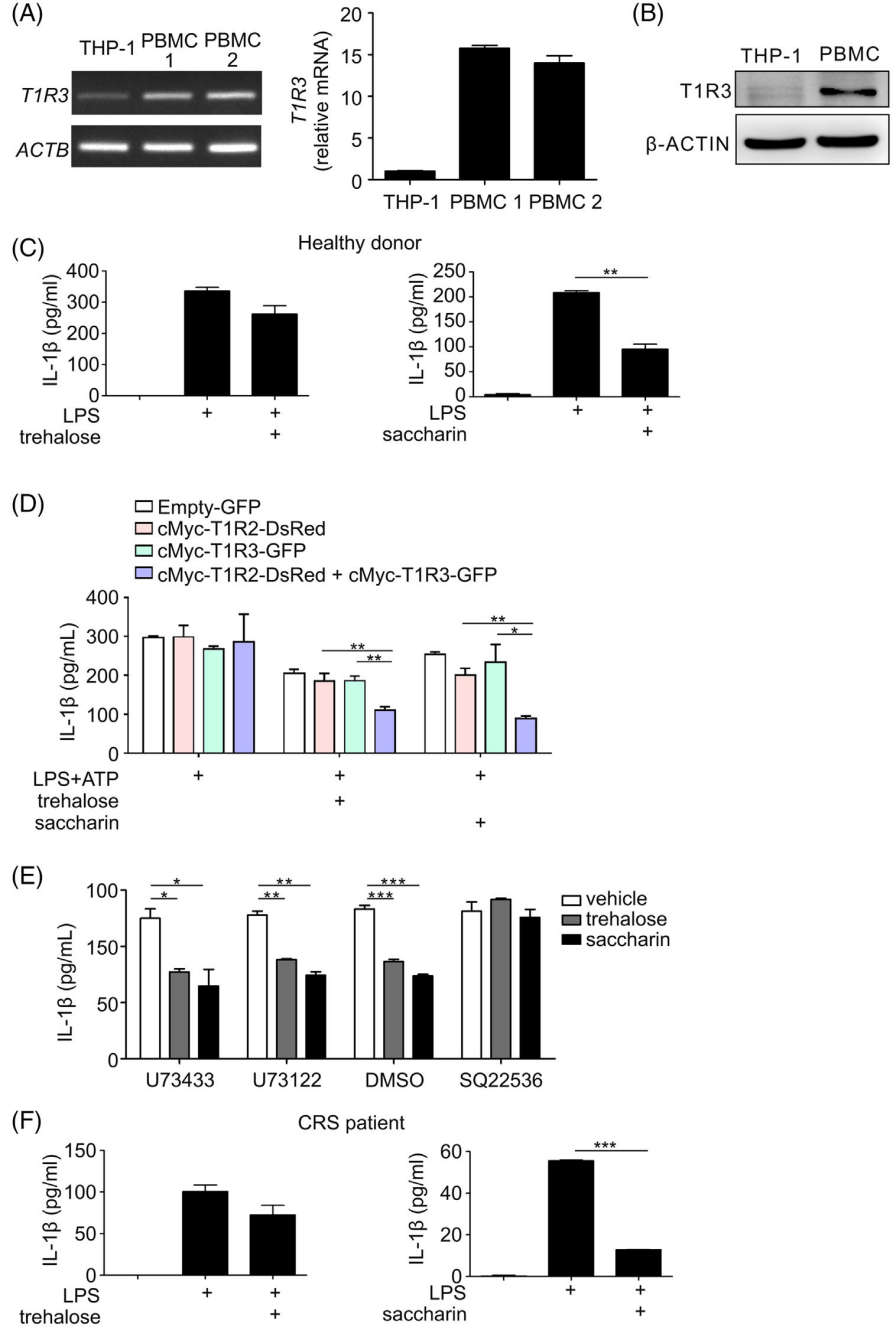
Human T1R3 in association with T1R2 contribute to the suppression of IL‐1β production. (A) Relative mRNA levels of T1R3 in THP‐1 cells or PBMC from two different donors were determined by using PCR (left) or qRT‐PCR (right) with normalisation to β‐actin mRNA. (B) Relative protein levels of T1R3 in THP‐1 cells or PBMC from healthy donor were determined by immunoblotting with β‐actin control. (C) Effect of trehalose (left) or saccharin (right) on the levels of IL‐1β in the supernatants of activated PBMC from healthy donors, as determined by ELISA. (D) Effect of T1R3 overexpression in the absence or the presence of T1R2 on trehalose (left)‐ or saccharin (right)‐mediated inhibition of IL‐1β levels in activated THP‐1 cells, as determined by ELISA. (E) Effect of U73122, U73433, and SQ22536 on trehalose‐ or saccharin‐mediated suppression of IL‐1β levels in activated THP‐1 cells, as determined by ELISA. (F) Effect of trehalose (left) or saccharin (right) on the levels of IL‐1β in the supernatants of activated PBMC from patient with chronic rhinosinusitis (CRS), as determined by ELISA. Data are expressed as means ± SDs. *P < .05, **P < .01, and ***P < .005, Student's *t*‐test. Data are representative of at least three independent experiments performed in duplicate or triplicate

Finally, we tested whether the findings obtained with mouse macrophages were also applicable to human macrophages. For this purpose, we first assessed the expression level of T1R3 mRNA in the human monocyte cell line THP‐1 and peripheral blood mononuclear cell (PBMCs) from two different donors. The expression level of T1R3 in PBMCs was relatively high compared to THP‐1 cells and varied somewhat amongst different donors (Figure 6A). The expression of T1R3 was also confirmed at the protein level (Figure 6B). Accordingly, PBMCs were used for assessing the effect of trehalose or saccharin on IL‐1β production. We found that the PBMC production of IL‐1β was moderately reduced by trehalose (25 mM) and significantly reduced by saccharin (0.5 mM) treatment (Figure 6C), supporting their anti‐inflammatory effect, particularly saccharin, in human PBMCs.

To further probe T1R3 function in human macrophages, we generated THP‐1 cells, with little intrinsic T1R3 expression, that overexpressed human T1R3 and human T1R2, either alone or in combination. THP‐1 cells were transduced with retroviruses encoding cMyc‐T1R3‐GFP, cMyc‐T1R2‐DsRed, or a combination of cMyc‐T1R2‐DsRed and cMyc‐T1R3‐GFP and then sorted for transgene expression based on coexpression of DsRed or GFP. The expression of T1R3 and T1R2 in the THP‐1 cells was confirmed by immunoblot analysis for human T1R3 and cMyc (Figure S11A). Of interest, surface expression of human T1R3 was barely detected in THP‐1 cells expressing T1R3 alone but abundant in THP‐1 cells coexpressing T1R2 and T1R3 (Figure S11B), consistent with a previous study.^47^ After macrophage differentiation with PMA treatment, these cells were stimulated in the presence of trehalose (10 mM) or saccharin (0.2 mM). Of note, IL‐1β production was significantly decreased by the expression of T1R3 combined with T1R2 but not T1R3 or T1R2 alone (Figure 6D), indicating the requirement of T1R2/T1R3 for effective response by trehalose and saccharin. T1R2/T1R3 is known to activate the downstream signalling via PLCβ2/IP3 and cAMP/PKA pathway.^48^ Thus, we next assessed the involvement of those pathways using an inhibitor of PLC activity (U73122) along with its inactive analogue (U73433) and an inhibitor of adenylyl cyclase (SQ22536) that inhibits cAMP production. Simultaneous exposure to SQ22536 but not U73122 was effective in reversing the inhibitory effects of trehalose and saccharin on IL‐1β production by THP‐1 cells (Figure 6E). These results suggest that STR agonists‐induced inhibitory effects on IL‐1β production is independent of PLC activity but relies on a mechanism involving cAMP production though the exact mechanism requires further investigation.

Furthermore, we evaluated the effect of trehalose and saccharin on IL‐1β production by PBMCs from a patient with CRS. As observed with PBMCs from healthy donors, trehalose (25 mM) treatment moderately and saccharin (0.5 mM) remarkably reduced the IL‐1β production by CRS PBMCs (Figure 6F). Collectively, these results suggest T1R3, likely in combination with T1R2, as a potential candidate implicated in the alleviation of IL‐1β production by trehalose and, particularly, saccharin, although we cannot rule out the possible contribution of other pathways.

## 3 DISCUSSION

**FIGURE 7:**
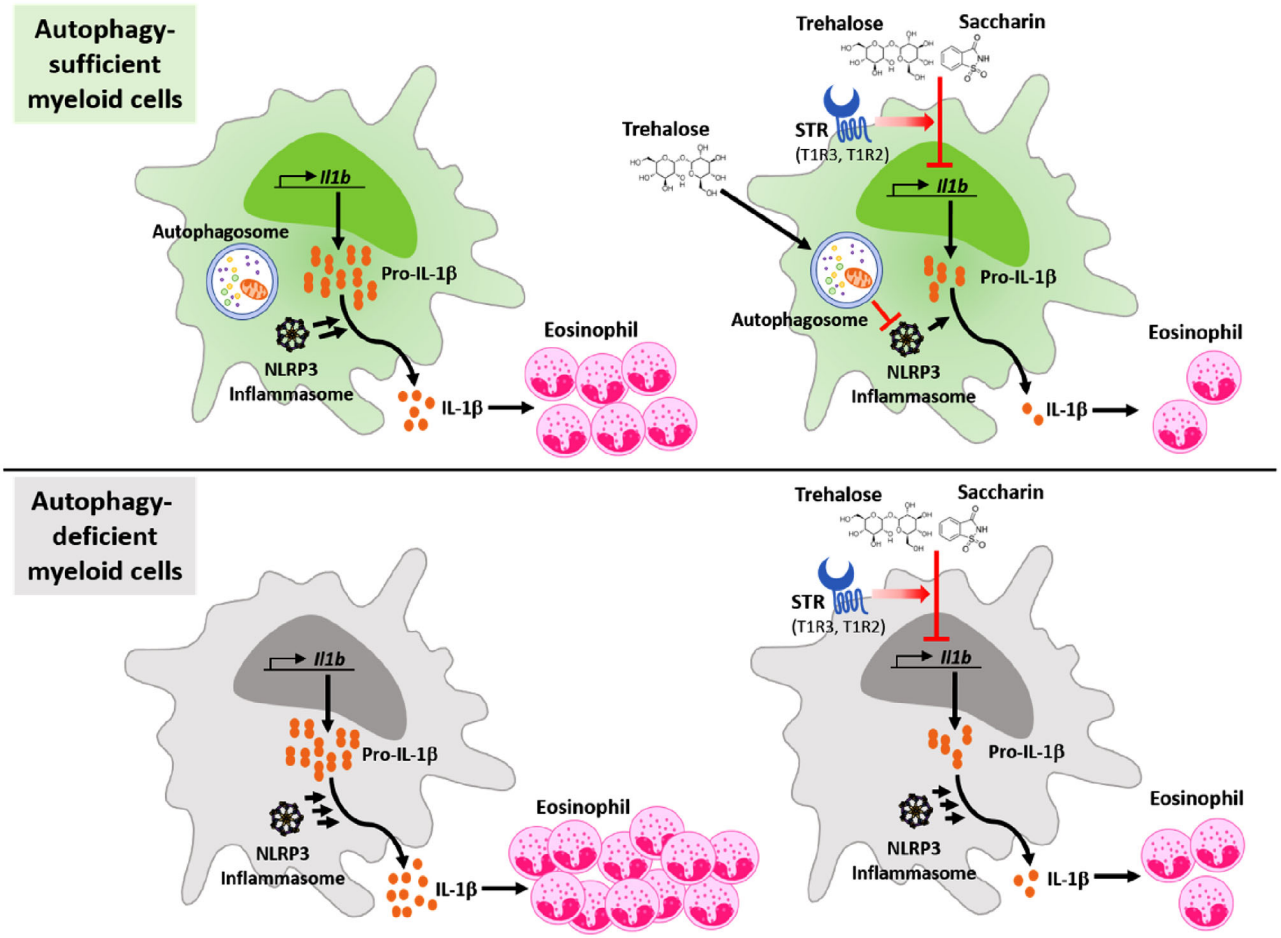
Proposed model of sweet taste receptor agonist‐mediated suppression of macrophage IL‐1β expression and alleviation of eosinophilic inflammation in chronic rhinosinusitis (CRS). Trehalose and saccharin‐attenuated eosinophilic inflammation linked to myeloid autophagy deficiency in a murine model of eosinophilic CRS (ECRS). Therapeutic efficacy was associated with their anti‐inflammatory effects, particularly IL‐1β production by macrophages. Thereby, activation of sweet taste receptor contributed to the alleviation of eosinophilic inflammation. Thus, sweet taste receptor agonists may provide a therapeutic option for eosinophilic inflammatory diseases including CRS.

In this study, we provide a new insight into the regulation of inflammatory cytokines and eosinophilic inflammation in CRS by STR agonists, particularly trehalose and saccharin. Based on our previous findings of a causal role of myeloid autophagy deficiency, especially macrophages, in ECRS, we presumed an enhancement of autophagy as an effective therapeutic strategy to treat eosinophilic inflammation in CRS. However, we unexpectedly found that trehalose, a natural sugar with pro‐autophagic activity, alleviates eosinophilic sinonasal inflammation and ECRS pathogenesis independently of autophagy via a preferential effect on the macrophage production of IL‐1β (Figure 7). In support, autophagy‐independent suppression of IL‐1β by trehalose was verified by assessing the production of various cytokines and the NLRP3 inflammasome pathway in autophagy‐deficient macrophages. Similar results were also found with diverse sweeteners, especially saccharin, in autophagy‐deficient macrophages and ECRS mice. The findings of a therapeutic effect of STR agonists on ECRS led to the identification of T1R3, likely in association with T1R2, as a potential contributor to such regulation. Moreover, the importance of this finding is supported by the STR agonist‐mediated suppression of IL‐1β production from human PBMCs and macrophage cells co‐expressing T1R2 and T1R3.

ECRS is a persistent sinonasal inflammatory condition attributed to dominant eosinophilia driven by Th2 cytokines (e.g., IL‐4, IL‐5, IL‐13).^49^ Due to its frequent association with refractory disease and NP recurrence following therapeutic intervention,^50^, ^51^ eosinophilia is an important target in CRS patients.^3^, ^9^ In general, ECRS is first treated with corticosteroids in an oral or topical route, which is frequently inefficacious and related to adverse systemic effects.^52^, ^53^, ^54^ Monoclonal biologics targeting T2 inflammation are increasingly used but only approved in patients with established T2 polyps due to challenges in identifying T2 inflammation in CRS without nasal polyps (CRSsNP) and in classifying distinct endotypes over variations in time, treatment and interpretation.^3^, ^55^ In this regard, our results of eosinophilia regulation by the STR agonists via a preferential effect on macrophage IL‐1β production may provide an alternative therapeutic modality to alleviate ECRS, particularly prior to its progression to severe and recurrent CRSwNP.

IL‐1β is produced principally from monocytes and macrophages and contributes to allergic disease by promoting mast cell activation and T2 cytokine production.^56^, ^57^ Supporting this, Th2 response and eosinophilic lung inflammation are significantly reduced in IL‐1β‐ and IL‐1R1‐deficient mice in a murine model of allergic asthma.^39^ Our previous study also demonstrated that depletion of macrophages overproducing IL‐1β or blockade of IL‐1R ameliorates CRS severity, mast cell infiltration, and eosinophilic inflammation related to myeloid cell‐specific autophagy deficiency.^21^ These results suggested IL‐1β as a major contributor to the pathogenesis of eosinophilic CRS linked to autophagy deficiency, though the potential contribution of IL‐1β‐independent mechanism cannot be excluded. Moreover, IL‐1β is known to drive type 1 and type 3 immune responses as well as type 2 inflammation.^58^ IL‐1β potentiates antigen‐specific responses of CD4+ T cells, supporting their expansion and commitment to Th1, Th2, and Th17 polarisation.^59^ Given the significant suppression of IL‐1β production by STR agonists, we speculated a broad effect of the STR agonists, especially trehalose and saccharin, on the diverse Th responses and inflammatory endotype‐related disorders including CRS. Supporting this, in our preliminary results, saccharin significantly alleviated imiquimod‐mediated psoriasis‐like skin inflammation in mice (data not shown), which is mediated by IL‐17 and neutrophils.^60^

It has been shown that trehalose has an anti‐inflammatory effect that inhibits cytokines production and protects against diverse stressors, including reactive oxygen species (ROS) and desiccation.^26^, ^29^, ^44^, ^61^ Previous studies demonstrated that trehalose could suppress the inflammatory responses of bone marrow cells including macrophages and attenuate inflammatory disorders related to septic shock, dye eye, kidney injury, and subarachnoid hemorrhage.^26^, ^27^, ^28^, ^29^, ^44^ As such, trehalose is considered a potential therapeutic for the treatment of oxidative damage and chronic inflammation, likely through an effect on autophagy induction.^23^, ^26^, ^28^, ^62^ Thus, we attempted to use trehalose in the treatment of ECRS caused by myeloid autophagy deficiency,^21^ although pathogenesis of CRS affected by autophagy and its dynamic regulation seems to be heterogeneous and context‐dependent^63^, ^64^, ^65^ and requires further investigation for therapeutic application.

In this study, we provided evidence that trehalose inhibits the inflammatory response of macrophages, particularly IL‐1β, and eosinophilic inflammation of CRS in an autophagy‐independent manner. In this respect, our results caution the prevailing view of autophagy regulation being the major mechanism underlying the anti‐inflammatory effect of trehalose. In addition, given trehalose as a naturally‐occurring sugar with less sweetness than sucrose, we found anti‐inflammatory effects of diverse STR agonists, especially saccharin, on macrophage IL‐1β production and eosinophilia in CRS regardless of dysfunctional autophagy. Saccharin is an artificial sweetener about 300 times sweeter than sucrose and is safe for human consumption with an acceptable daily intake of 5 mg/kg bw/d.^66^ The anti‐inflammatory effect of saccharin, as demonstrated here, is supported by previous studies showing the saccharin‐mediated inhibition of iNOS expression in RAW264.7 macrophage cells^67^ and of mRNA expression of cytokines, including IL‐1β, in 3T3‐L1 adipocyte cells.^68^

Of interest is the finding of the involvement of T1R3 in mediating the effects of trehalose and saccharin on the regulation of macrophage IL‐1β and eosinophilic inflammation in CRS. T1Rs are evolutionally conserved proteins that act as nutrient sensors and are expressed in taste cells but also in diverse tissues such as gastrointestinal tract, pancreas, adipose tissues, bone and immune system.^44^, ^69^, ^70^, ^71^ Genetic analysis of sweet tasting identified a single principal locus in mice, named *Sac*, affecting the responses to multiple sweet substances including saccharin,^72^, ^73^ which led to the discovery of the T1R‐related gene, *T1R3*.^41^, ^74^ In general, T1R3 associates with T1R2 to form a functional STR, whereas the heterodimer of T1R1 and T1R3 form a umami taste receptor that responds to most L‐amino acids.^40^, ^69^ Although the biological role of STR in extra‐oral tissues remains unclear, the broad distribution of ectopic STR suggests diverse functions beyond sweet taste perception.^69^ Of interest, STR is coexpressed with bitter taste receptor T2Rs in solitary chemosensory cells, rare chemosensing epithelial cells, of sinonasal cavity of CRS patients and suppresses T2R‐mediated release of antimicrobial peptides from the surrounding epithelial cells during sinonasal bacterial infection,^75^, ^76^ implicating STR in the inhibition of immune responses. STR was also found in murine macrophages^44^, ^77^ and diverse human immune cells, including monocytes, PMN, B, and T cells,^71^ and saccharin‐modulated PMN chemotaxis via T1R2 and T1R3 but not T1R1.^71^ Here, we demonstrated the direct contribution of STR, especially T1R3, to the regulation of macrophage IL‐1β production and eosinophilia in CRS using gurmarin, T1R3 deletion, and STR overexpression. This finding was also supported by our results of macrophage treatment with L‐serine that signals via T1R1/T1R3 and is compatible with the anti‐inflammatory effects of L‐serine on neuroinflammation after brain injury.^46^, ^78^ Although significant, the partial involvement of T1R3 in this context, especially trehalose at high concentrations (data not shown), might be attributed to a low expression of ectopic STR in macrophages relative to taste cells, the ROS scavenging effect of trehalose,^79^ or the contribution of other cognate receptors for saccharin such as T2Rs and TRPV1.^80^, ^81^ In this regard, it merits further study using T1R3 knockout mice to confirm our results and the involvement of other regulatory mechanism(s).

A recent study reported that, compared with other locations (inferior turbinate, middle turbinate, septum), T1R3 is highly expressed in the ethmoid sinus from patients with CRS,^82^ a sinonasal subsite typically affected by acute rhinosinusitis and early CRS. Moreover, T1R3 expression is also upregulated in the nasal mucosa of children with CRS,^83^ and an increased intensity of sweet taste but decreased bitter sensitivity is observed in adult patients with CRSwNP.^84^ Although it requires to clarify the types of cells expressing T1R3 in the sinonasal mucosa, these findings thus suggest a potential role of T1R3 in the pathophysiology of CRS, and the clinical relevance of its modulation by STR agonists, likely in patients with CRS unrelated to bacterial rhinosinusitis, awaits further study.

## 4 MATERIALS AND METHODS

### 4.1 Mouse

All mouse experiments were approved by Institutional animal care and use committee (IACUC) of Asan Institute for Life Sciences for Animal Care and Use. *Atg7* ^fl/fl^;*Lyz2*‐Cre mice with myeloid cell‐specific deletion of *Atg7* were generated by crossing *Lyz2*‐Cre mice (Jackson Laboratories, stock number 4781) with *Atg7* ^fl/fl^ mice (kind gift of Masaaki Komatsu, Juntendo University, Tokyo, Japan). All experiments were performed using littermates. C57BL/6 mice were obtained from Koatech (South Korea).

### 4.2 Reagent

Trehalose (T9531), sucralose (69293), saccharin (S1002), glucose (G8270), sucrose (S0389), maltose (M5885), lactose (L254) and L‐serine (S4311) were obtained from Sigma–Aldrich. Acesulfame K was obtained from Tokyo Chem. Industry (A1490). U73122 (1268) and its inactive analogue U73433 (4133), and adenylyl cyclase inhibitor SQ22536 (1435) were from TOCRIS. Recombinant mouse M‐CSF was purchased from Peprotech (315‐02).

### 4.3 Murine chronic rhinosinusitis model

To induce CRS with eosinophilic inflammation, mouse was intranasally challenged with combination of ovalbumin (Sigma–Aldrich, A5503) and *Aspergillus oryzae* protease (Sigma–Aldrich, P6110) three times per week for a total of 5 weeks, as described.^31^ Control mouse was intranasally treated with DPBS. For assessing the effect of trehalose (Sigma–Aldrich, T9531) or saccharin (Sigma–Aldrich, S1002), mouse was administered trehalose (100 mg/kg for intranasal or 2 g/kg for intraperitoneal injection) or saccharin (2 mg/kg for intranasal administration) 5 h before intranasal challenge with combination of ovalbumin and protease 3 times per week for 5 weeks. For blockade of the T1R3 receptor, mouse was given an intranasal administration of gurmarin (Peptides International, GUR‐3810‐PI; 1 mg/kg) 1 h before each trehalose or saccharin administration. After CRS development, the nasal tissue from CRS mice was harvested and subject to fixation in 4% formaldehyde. Each tissue was paraffin‐embedded, sectioned at 3 μm, and examined in a blinded manner for group assignment by a pathologist of Asan Medical Center.

### 4.4 Differential cell counting

Cardiac puncture was used to collect blood samples, and the numbers of different cell types were measured with ADVIA hematology system (Bayer HealthCare) and examined by pathologist of Asan Medical Center.

### 4.5 Histologic analysis

For measurement of maximal mucosal thickness and epithelial hyperplasia, sections were stained using hematoxylin and eosin (H&E) and analysed in a blinded manner for group assignment by pathologist of Asan Medical Center. Epithelial hyperplasia was scored on none (0) to severe (4) basis, as described.^85^ Maximal mucosal thickness was assessed at the transition zone between respiratory and olfactory epithelium (CellSens analysis software, Olympus). Eosinophils were analysed after staining with Sirius Direct Red 80 (Sigma–Aldrich, 365548), and eosinophil infiltration into the sinonasal mucosal was measured by counting the cell number per high‐powered field, as previously described.^31^ Mast cells were analysed after acidic toluidine blue staining, as described.^21^

### 4.6 Immunofluorescence

To detect IL‐1β‐producing macrophages in sinonasal mucosa of CRS mice, slide was incubated with primary antibody specific for CD68 (AbD Serotec, MCA1957; 1:100) and IL‐1β (Cell Signaling Technology, 12242; 1:100) for 1 h, and then goat anti‐rat F(ab′)2‐Alexa Fluor 647 (Jackson ImmunoResearch, 712‐606‐153; 1:250) and goat anti‐mouse F(ab′)2‐Alexa Fluor 488 (Jackson ImmunoResearch, 705‐546‐147; 1:250) in PBS with 1% goat serum (Invitrogen, 31872) and 1% bovine serum albumin (Sigma–Aldrich, A9647) for 30 min. Autophagy induction was detected using rabbit anti‐mouse LC3B antibody (Novus, NB600‐1384; 1:400) along with rat anti‐mouse CD68 antibody (Bio‐rad, MCA1957; 1:100), followed by goat anti‐rabbit F(ab′)2‐Alexa Fluor 647 (Jackson ImmunoResearch, 111‐546‐143; 1:250) and goat anti‐rat F(ab′)2‐Alexa Fluor 488 (Jackson ImmunoResearch, 111‐606‐144; 1:250). Image was analysed by lymphocyte separation medium (LSM) 710 confocal microscope (Carl Zeiss) after mounting of slide with ProLong Gold (Molecular Probes, P36930).

### 4.7 Macrophage isolation and culture

PMs were isolated from peritoneal cavity of 8‐ to 10‐week‐old mice following injection of 3.85% thioglycolate media intraperitoneally (BD Biosciences, 211260), as described.^86^ Macrophages collected from peritoneal cavity were seeded in a culture plate and then incubated for 3 h. Non‐adherent cells were discarded by Dulbecco's modified eagle medium (DMEM) washing, and the adherent macrophages were used for the indicated assays. To prepare primary BMMs, bone marrow cells were isolated from tibia and femur of 8‐ to 10‐week‐old mice, as described.^86^ Cells were incubated in DMEM with 10% fetal bovine serum (FBS), 2 mM glutamine, 100 μg/ml streptomycin, and 100 U/ml penicillin for 6 h. Non‐adherent cells were collected and cultured in the same media containing M‐CSF (Peprotech, 315‐02; 20 ng/ml) for 6 days with 2 days interval of media change. Thereafter, BMMs were harvested after DPBS washing and were used for further experiments.

### 4.8 HL‐60 cell culture and stimulation

For assessing the effect of trehalose on eosinophil generation of Th2 cytokines, a cell line model of human eosinophilic HL‐60 cells that typically express IL‐5 receptor was used.^87^, ^88^ HL‐60 cells clone 15 (5 × 10^5^ cells/ml) were cultured in fresh RPMI‐1640 (Gibco, 11875‐093) with 10% FBS (Gibco, 16000‐044), buffered with 25 mM 4‐(2‐hydroxyethyl)‐1‐piperazinepropanesulfonic acid (EPPS), and then subject to eosinophilic differentiation by adding 0.5 mM sodium butyrate at 37°C for 5 days, as previously described.^89^ Differentiated HL‐60 cells were pretreated with 25 mM trehalose for 15 min and thereafter stimulated with LPS (10 ng/ml) for 24 h for further analysis.

### 4.9 Quantitative real‐time RT‐PCR

Turbinate and septal mucosa were collected and incubated in RNAlater solution (Invitrogen, AM7020) for the extraction of RNA. RNA isolation was performed with the RNeasy Mini Kit (Qiagen GmbH, 74134) according to manufacturers’ instructions. RNA samples prepared from mouse tongue, PM, BMM, human PBMC and THP‐1 cells were also purified. One microgram of total RNA was used to synthesise cDNA with the ReverTra Ace qPCR RT kit (Toyobo, FSQ‐101). qRT‐PCR was conducted using SYBR Green RT‐PCR master mix (Toyobo, QPK‐201) and analysed with LightCycler 480 RT‐PCR system (Roche Diagnostics, Basel, Switzerland). For analysing trehalose and saccharin effect on the expression of inflammatory cytokines, macrophages were pretreated with trehalose or saccharin for 30 min before stimulation with LPS (InvivoGen, tlrl‐eklps; 10 ng/mL) for 1 h (trehalose) or 2 h (saccharin). The relative mRNA levels of mouse *Ilb*, *Tnfa*, *Il6*, *Il4*, *Il5*, *Il13*, *Ccl5*, *and Ccl11* determined by qRT‐PCR. The following primer sequences were used:

Actb
: 5′‐GGCTGTATTCCCCTCCATCG‐3′ (forward) and 5′‐CCAGTTGGTAACAATGCCATGT‐3′ (reverse),
Il1b
: 5′‐GAATGACCTGTTCTTTGAAGT‐3′ (forward) and 5′‐TTTGTTGTTCATCTCGGAGCC‐3′ (reverse),
Tnfa
: 5′‐CCTGTAGCCCACGTCGTAGC‐3′ (forward) and 5′‐TTGACCTCAGCGCTGAGTTG‐3′ (reverse),
Il6
: 5′‐TGGAGTCACAGAAGGAGTGGCTAAG‐3′ (forward) and 5′‐TCTGACCACAGTGAGGAATGTCCAC‐3′ (reverse),
Il4
: 5′‐CATGGGCATTTTGAACGAGGTCA‐3′ (forward) and 5′‐CTTATCGATGAATCCAGGCATCG‐3′ (reverse),
Il5
: 5′‐GCAATGAGACGATGAGGCTTC‐3′ (forward) and 5′‐GCCCCTGAAAGATTTCTCCAATG‐3′ (reverse),
Il13
: 5′‐AGACCAGACTCCCCTGTGCA‐3′ (forward) and 5′‐TGGGTCCTGTAGATGGCATTG‐3′ (reverse),
Ccl5
: 5′‐AGATCTCTGCAGCTGCCCTCA‐3′ (forward) and 5′‐GGAGCACTTGCTGCTGGTGTAG‐3′ (reverse),
Ccl11
: 5′‐GAATCACCAACAACAGATGCAC‐3′ (forward) and 5′‐ATCCTGGACCCACTTCTTCTT‐3′ (reverse),
T1r3
: 5′‐CAGGCAGTTGTGACTCTGTTG‐3′ (forward) and 5′‐TGCGATGCAGATACCTCGTG‐3′ (reverse).

Gene expression level was normaliaed by β‐actin and was calculated relative to the expression in control group. The relative mRNA levels of human *T1R3*, *IL5*, *IL5R*, and *IL13* were assessed using qRT‐PCR with the following primer sequences.

ACTB
: 5′‐GCATCGTCACCAACTGGGAC‐ 3′ (forward) and 5′‐GGTCTCAAACATGATCTGGG‐3′ (reverse),
T1R3
: 5′‐CCGCCTACTGCAACTACACG‐3′ (forward) and 5′‐CTAGCACCGTAGCTGACCTG‐3′ (reverse).
IL5
: 5′‐TGGAGCTGCCTACGTGTATG‐3′ (forward) and 5′‐TTCGATGAGTAGAAAGCAGTGC‐3′ (reverse)
IL5R
: 5′‐ATCATCGTGGCGCATGTATTAC‐3′ (forward) and 5′‐AAAGAACTTGAGCCAAACCAGT‐3′ (reverse)
IL13
: 5′‐ CCTCATGGCGCTTTTGTTGAC ‐3′ (forward) and 5′‐TCTGGTTCTGGGTGATGTTGA‐3′ (reverse).

Gene expression level normalised by β‐actin in the experimental group was calculated relative to the expression in the control group.

### 4.10 Cytokine measurement by ELISA

Peritoneal or BMMs were pretreated with trehalose, saccharin or the indicated sugars 15 min prior to stimulation with LPS (Invivogen, tlrl‐eklps; 10 ng/ml) for 8 h to measure IL‐6 and TNF‐α release, or for 4 h followed by adding ATP (Sigma–Aldrich, A6419; 5 mM) for IL‐1β measurement. To determine the effect of gurmarin, gurmarin (Peptides international, GUR‐3810‐PI) was added 15 min prior to the pretreatment with trehalose or saccharin. Levels of inflammatory cytokines in culture supernatant were measured by mouse duoset ELISA kits for IL‐1β (DY401), IL‐6 (DY406), TNF‐α (DY410), and human duoset ELISA kits for IL‐1β (DY201). All ELISA kits were obtained from R&D systems.

### 4.11 Immunoblot analysis of NLRP3 inflammasome, Atg7 protein, and human T1R3

To analyse the expression of NLRP3 inflammasome, primary PMs were incubated for 3 h after seeding in the culture plate. Macrophages were assayed in opti‐MEM media (Gibco, 31985) in the presence or the absence of trehalose or saccharin 15 min prior to LPS stimulation (Invivogen, tlrl‐eklps; 10 ng/ml) for 3 h followed by adding ATP (Sigma–Aldrich, A6419; 5 mM) for 30 min to release IL‐1β. To confirm Atg7 protein deficiency in macrophage of *Atg7* ^fl/fl^ or *Atg7* ^fl/fl^;*Lyz2*‐Cre mice, PMs were harvested, seeded, incubated for 3 h, and the adherent macrophages were collected for further analysis. Human T1R3 was analysed from cell lysates obtained from THP‐1 cell line or PBMCs, as described.^86^ Proteins in the culture supernatant were concentrated by chloroform‐methanol precipitation, as described previously.^90^ Primary antibodies used were goat anti‐mouse IL‐1β antibody (R&D Systems, AF‐401‐NA; 1:1000), mouse monoclonal anti‐NLRP3 antibody (Adipogen, AG‐20B‐0014; 1:1000), mouse monoclonal anti‐caspase‐1 antibody (AdipoGen, AG‐20B‐0042; 1:1000), rabbit anti‐Atg7 antibody (Cell Signaling, 2631; 1:1000), rabbit anti‐human T1R3 (abcam, ab229015; 1:1000), and mouse anti‐β‐Actin antibody (BD Biosciences, 612657; 1:5000).

### 4.12 RNA interference

BMMs were transfected with siRNA (600 pmoles) using Amaxa system (Nucleofector II). BMMs (3×10^6^ cells) were resuspended in Amaxa solution T (Lonza, VVCA‐1002; 100 μl), admixed with siRNA, and then transfected under program T‐20. Cells were seeded, incubated in the culture media with M‐CSF (20 ng/ml) for 16 h, and thereafter subject to a media exchange for 32 h. After incubation, BMMs were assayed in opti‐MEM media (Gibco, 31985) in the absence or the presence of trehalose or saccharin prior to LPS (Invivogen, tlrl‐eklps; 10 ng/ml) stimulation for 3 h followed by adding ATP (Sigma–Aldrich, A6419; 5 mM) for 30 min to secrete IL‐1β. IL‐1β amount secreted into the supernatants of BMMs was determined by ELISA. The siRNA targeting mouse T1R3 was purchased from Dharmacon (M‐046255‐01‐0005). For efficiency check, total RNA was prepared and then subjected to qRT‐PCR analysis.

### 4.13 T1R3 deletion by CRISPR/Cas9‐RNP

sgRNA target sites were designed using the web programs Cas‐Design (http://www.rgenome.net/). sgRNA target for T1r3 sequences are 5′‐CTGGCACTATAGCTGACCTG‐3′ for sgRNA #1 and 5′‐CCATGGCCAGGAACAAACCA‐3′ for sgRNA #2. Target sequences were selected that had a PAM (NGG) sequence, high out‐of‐frame score, and no off‐target sites in the genome. sgRNAs were synthesised with 2′‐O‐methyl 3′ phosphorothioate modification in the first and last three nucleotides (SYNTHEGO). To generate T1R3 knock‐out cells, BMMs (5×10^6^ cells) were resuspended in Amaxa solution T (Lonza, VVCA‐1002; 100 μl) with a mixture of RNP including 500 pmoles of sgRNA, Cas9 (IDT, 1081061) and Enhancer (IDT, 1075915), and then transfected under program T‐20 Amaxa Nucleofector II system. Cells were then seeded, incubated in the culture media with M‐CSF (20 ng/ml) for 24 h, and thereafter subject to media exchange for 48 h. After incubation, BMMs were assayed in opti‐MEM media in the absence or the presence of trehalose or saccharin prior to LPS stimulation for 3h followed by adding ATP (5 mM) for 30 min to secrete IL‐1β. IL‐1β amount secreted into the supernatants of BMMs was assessed by ELISA. Efficiency of T1R3 deletion in BMM was performed by T7E1 cleavage assay using guide‐it^TM^ mutation detection kit (TAKARA, 631448) and was calculated using the cleaved DNA ratio by 100 × (intensity of fragment 1 + intensity of fragment 2)/(intensity of parent fragment + intensity of fragment 1 + intensity of fragment2) (Biosearch Technology). The following primers were used for detection of *T1r3* DNA cleaved ratio; 5′‐AACCAGAAGAACACAACCCAAC‐3′ (forward) and 5′‐CAGTTCCAGCTGAAGTTCTGC‐3′ (reverse).

### 4.14 Detection of CD69 expression on blood eosinophils

Mice were administered with or without 5 mg/kg LPS in the absence or the presence of intraperitoneal trehalose (40 mg/kg) injection. After 4 h, leukocytes in each group of mice were obtained from the peripheral blood after lysing red blood cells. Then, cells were mixed with Fcγ receptor‐binding inhibitor (eBioscience, 553146) at 4°C for 15 min and stained with antibodies against rat anti‐mouse Siglec F‐PE (eBioscience, 552123), rat anti‐mouse CCR3‐FITC (R&D systems, FAB729F), and Armenian hamster anti‐mouse CD69‐APC (Biolegend, 104513) for 1 h at 4°C. CD69 expression on Siglec F+CCR3+ eosinophils was assessed by FACS analysis using FACS Accuri 6 (BD Biosciences).

### 4.15 PBMC isolation and stimulation

The human study was approved by the Asan Medical Center Institutional Review Board (Seoul, Korea) with a written informed consent obtained (approval numbers: S2011‐0384). Normal healthy donors or CRS patients receiving endoscopic sinus surgery at the Asan Medical Center were enrolled from 2011 to 2016. PBMCs were isolated from whole blood by density gradient centrifugation (LSM, MP Biomedicals, 50494). For stimulation, PBMCs were incubated for 1 h to recover, pretreated with trehalose or saccharin 15 min prior to LPS stimulation (100 ng/ml) for 4 h. IL‐1β amount secreted into the supernatants of PBMC was assessed by ELISA.

### 4.16 Generation of THP‐1 cells overexpressing T1R3

Plasmid for cMyc‐human T1R3 (113949, Erik Procko) and cMyc‐human T1R2 (113946, Erik Procko) were purchased from Addgene, cloned into the pMX‐IRES‐EGFP and pMX‐IRES‐DsRed retroviral vector, respectively. For transduction, Plat A cells were transfected with each retroviral vector (pMX‐IRES‐EGFP, pMX‐IRES‐cMyc‐T1R3‐EGFP and pMX‐IRES‐cMyc‐T1R2‐DsRed) using X‐tremeGENE 9 (Roche, 50730400). After 24 h of media change, virus‐containing supernatant was harvested for 24 h, mixed 1:1 ratio with fresh RPMI1640 (Gibco, 11875‐093) medium containing polybrene (10 μg/ml), and then added into THP‐1 cells for virus transduction for 6 h. Thereafter, cells were washed, incubated in fresh RPMI1640 with 10% FBS and 500 μM of β‐mercaptoethanol (Gibco, 21985) for 3 days, and then sorted by a FACSAria III cell sorter (BD Biosciences) based on GFP or DsRed expression. T1R3 and cMyc expression levels were detected by immunoblot analysis using rabbit anti‐human T1R3 antibody (Abcam, ab229015; 1:1000) and anti‐cMyc antibody (Biolegend, 626802; 1:1000). To detect surface expression of T1R3, THP‐1 cells were stained with anti‐cMyc antibody‐Alexa Fluor 647 (Cell Signaling Technology, 2233s; 1:50) and measured by Accuri C6 flow cytometer (BD Biosciences). FlowJo software (Tree Star, Ashland, OR) was used to analyse data.

THP‐1 cells were stimulated with phorbol 12‐myristate 13‐acetate (PMA, Sigma–Aldrich, P1585; 10 ng/ml) in RPMI1640 containing 10% FBS and 500 μM of β‐mercaptoethanol for 48 h. After PMA stimulation, THP‐1 cells were washed by opti‐MEM, and then pretreated with trehalose or saccharin 15 min before LPS (10 ng/ml) stimulation for 3 h, followed by adding ATP (5 mM) for 30 min. The production level of IL‐1β was assessed by ELISA.

### 4.17 Statistical analysis

All data analysis was performed using GraphPad Prism version 5.0 software. Statistical comparison amongst three or more groups was conducted using one‐way analysis of variance (ANOVA) test with Dunnet correction. Statistical comparison between two groups was performed by two‐tailed Student *t*‐test or Mann–Whitney *U*‐test. A *P*‐value of less than .05 was considered to be statistically significant.

## CONFLICT OF INTEREST

The authors declare that there is no conflict of interest that could be perceived as prejudicing the impartiality of the research reported.

## Supporting information

Supporting Information
