# Supplementary material for: Sweet taste receptor agonists attenuate macrophage IL‐1β expression and eosinophilic inflammation linked to autophagy deficiency in myeloid cells
Source: Clin Transl Med. 2022 Aug 21;12(8):e1021. doi: 10.1002/ctm2.1021 (PMC9393075; doi:10.1002/ctm2.1021)
Supplement: Supplementary file 1 — Supporting Information [file CTM2-12-e1021-s001.docx]

**
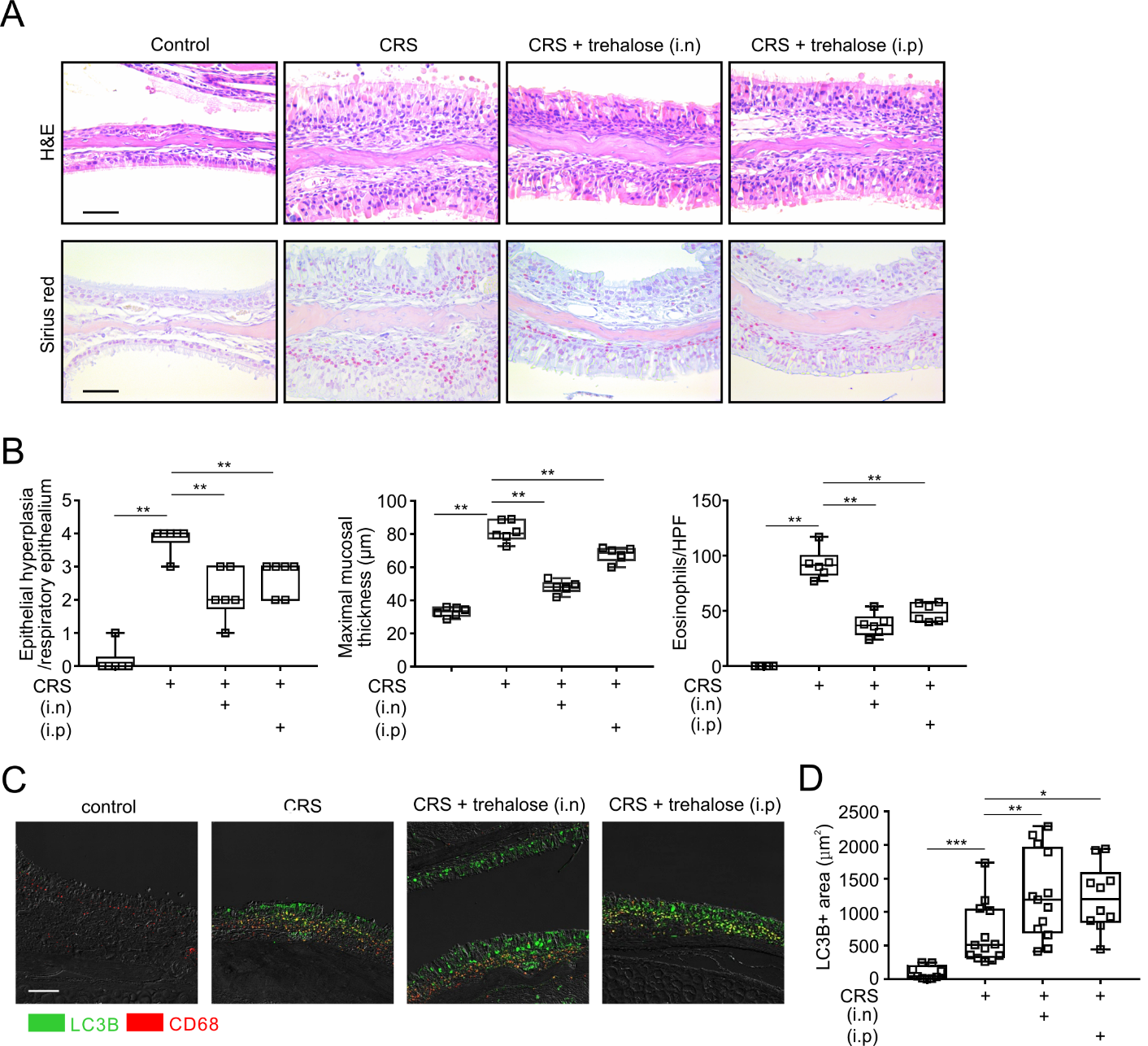
**

**Figure S1.** Trehalose ameliorates eosinophilic inflammation in wild-type mice with ECRS.

(A) Effect of trehalose via intranasal (i.n.) or intraperitoneal (i.p.) injection on murine model of ECRS. Representative photographs of hematoxylin and eosin (H&E; *upper*)- and Sirius red (*lower*)-stained tissue sections. (B) Scores of epithelial hyperplasia, maximal mucosal thickness in H&E-stained tissue sections, and Sirius red-positive eosinophil counts of the lamina propria in Sirius red-stained tissue sections. (C) Representative dual-immunofluorescence staining for LC3B and CD68+ macrophages in the sinonasal tissue for each group of mice is shown. Yellow signals indicate colocalization. (D) The statistical bar chart shows the area of LC3B. Scale bars = 50 μm. Data are expressed as box-and-whisker plot with the box marking 25th, median, and 75th percentiles (*n* = 6 per group). **P* < .05, ***P* < .01, Mann-Whitney *U* test.


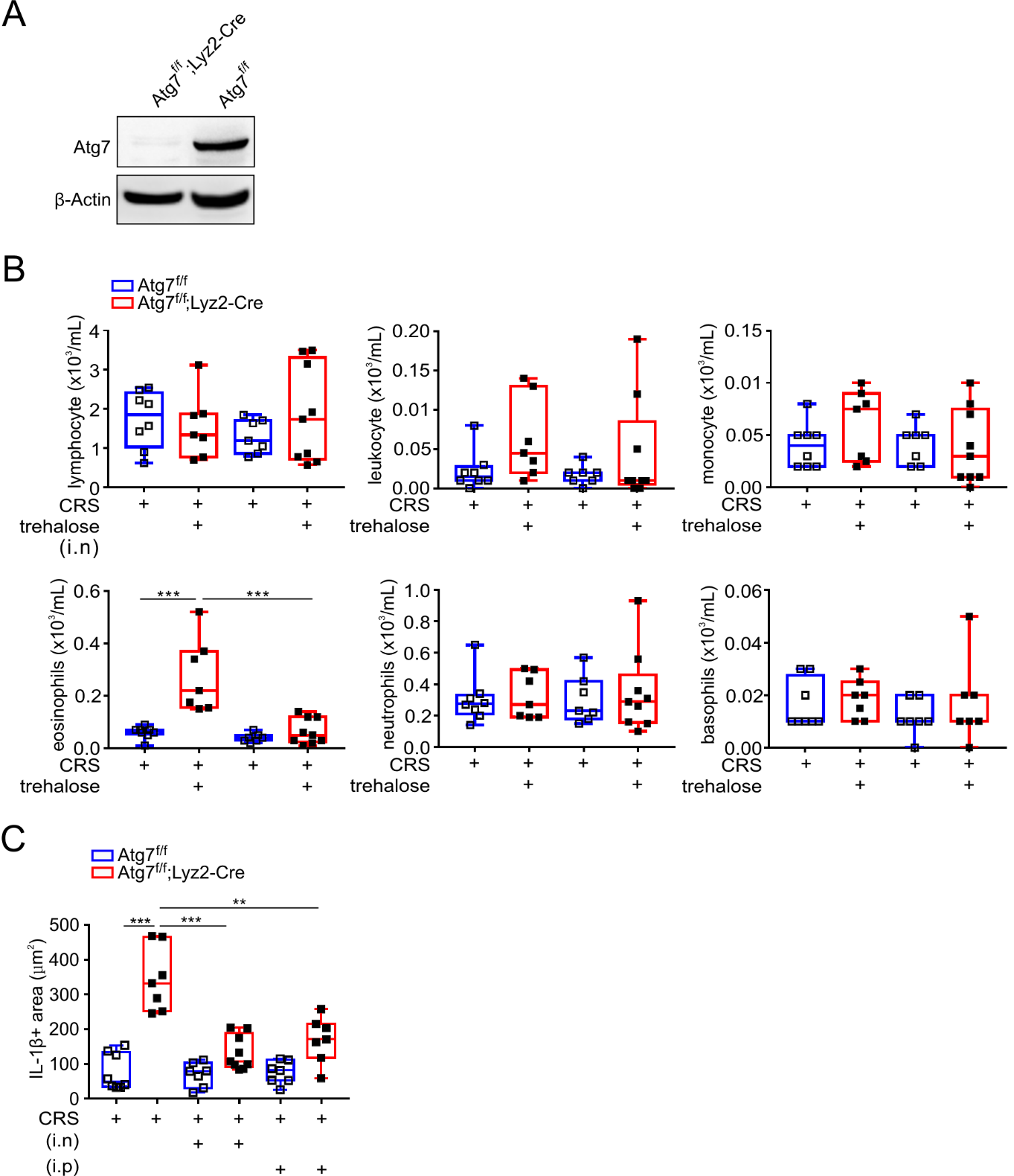


**Figure S2.** Trehalose alleviates blood eosinophilia and IL-1β levels in CRS.

(A) Lysates of macrophages from *Atg7^fl/fl^* or *Atg7^fl/fl^*;Lyz2-Cre mice were immunoblotted for Atg7 and actin. (B) Effect of trehalose via intranasal (i.n.) injection on lymphocyte, leukocyte, monocyte, eosinophil, neutrophil, and basophil counts in blood from each group of mice. (C) Statistical bar chart shows the area of IL-1β in the sinonasal tissue from each group of mice. Data are expressed as box-and-whisker plot with the box marking 25th, median, and 75th percentiles (*n* = 7-9 per group). ***P* < .01, ****P* < .005, Mann-Whitney *U* test.


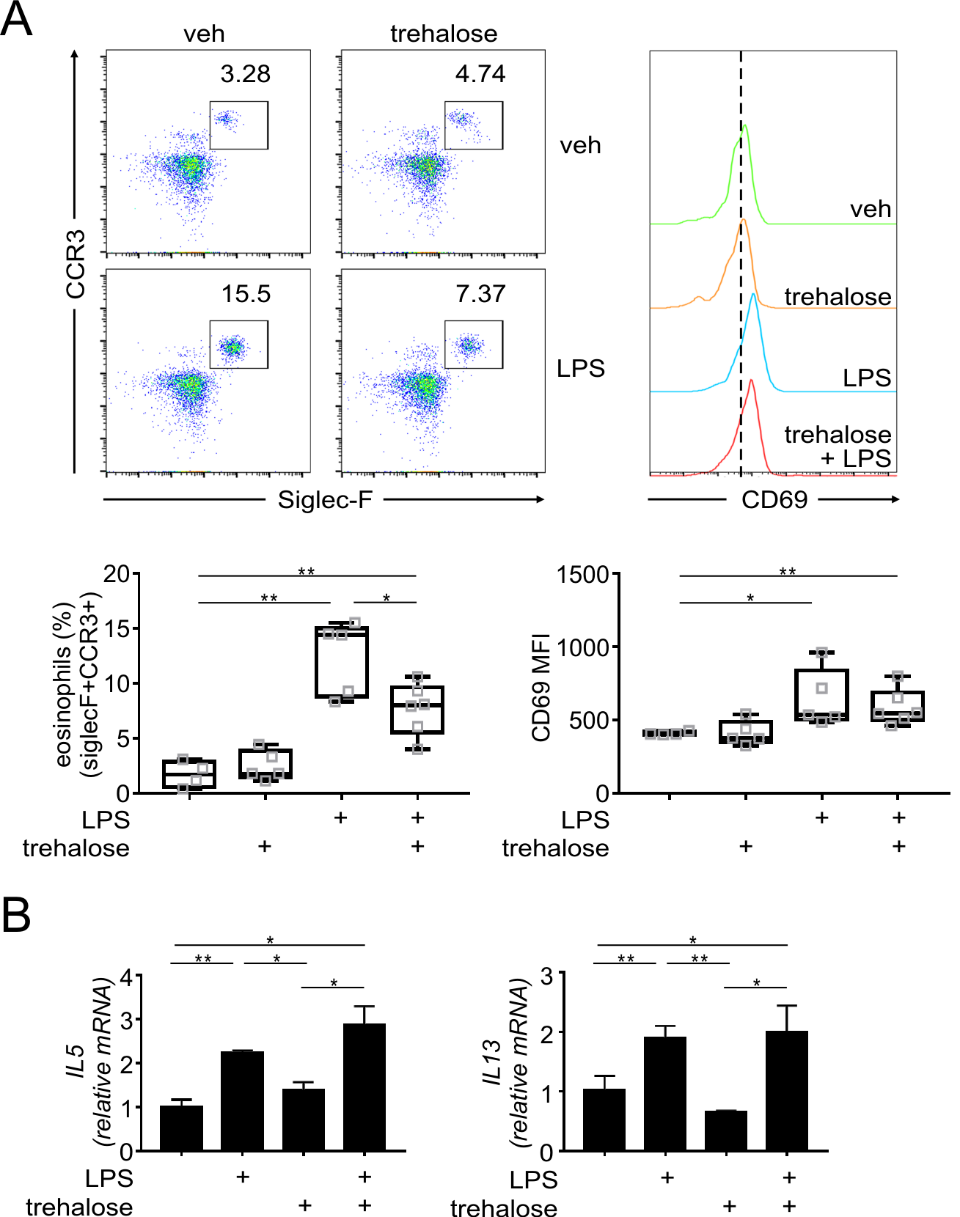


**Figure S3.** Effect of trehalose on eosinophil expression of CD69 and IL-5 and IL-13.

(A) The presence of eosinophils was determined by analyzing siglec-F expression on CCR3+ cells in peripheral blood from mice treated with LPS. Representative FACS profile (*upper left*) and statistical bar chart (*lower left*) showing the percentages of siglec-F+CCR3+ eosinophils. Representative FACS profile (*upper right*) and statistical bar chart (*lower right*) show surface expression of CD69 on Siglec-F+CCR3+ eosinophils. Data are expressed as box-and-whisker plot with the box marking 25th, median, and 75th percentiles (*n* = 4-6 per group). (B) Effect of trehalose on relative mRNA levels of IL-5 and IL-13 in human eosinophilic HL-60 cells treated with LPS was determined by using qRT-PCR and normalized to β-actin mRNA. Data are expressed as means ± SDs. Data are representative of at least three independent experiments performed in duplicate or triplicate. **P* < .05, ***P* < .01, Student’s *t*-test or one-way ANOVA.





**Figure S4.** Trehalose suppresses inflammatory cytokine production from autophagy-deficient macrophages.

Dose-dependent effect of trehalose on the levels of IL-1β (*top*), IL-6 (*middle*), and TNF-α (*bottom*) in culture supernatants of activated peritoneal macrophages from *Atg7^fl/fl^* or *Atg7^fl/fl^;Lyz2*-Cre mice, as determined by ELISA. Data are expressed as means ± SDs. **P* < .05, ***P* < .01, ****P* < .005, and ###*P* < .005, One-way ANOVA.





**Figure S5.** Treatment with saccharin reduces inflammatory cytokine production from autophagy-deficient macrophages.

Dose-dependent effect of saccharin on the levels of IL-1β (*left*), IL-6 (*middle*), and TNF-α (*right*) in culture supernatants of activated peritoneal macrophages from *Atg7^fl/fl^* or *Atg7^fl/fl^;Lyz2*-Cre mice, as determined by ELISA. Data are expressed as means ± SDs. **P* < .05, ***P* < .01, ****P* < .005, ##*P* < .01, and ###*P* < .005, One-way ANOVA.





**Figure S6.** L-serine recapitulates the anti-inflammatory effect of trehalose and saccharin.

Effect of trehalose, saccharin, or L-serine on the levels of IL-1β (*left*) and TNF-α (*right*) in culture supernatants of activated peritoneal macrophages from *Atg7^fl/fl^* or *Atg7^fl/fl^;Lyz2*-Cre mice were determined by ELISA. Data are expressed as means ± SDs. ****P* < .005, ###*P* < .005, Student’s *t*-test.





**Figure S7.** Trehalose and saccharin preferentially suppress IL-1β production from autophagy-deficient BMM. Effect of trehalose or saccharin on the levels of IL-1β (*left*) and TNF-α (*right*) in culture supernatants of activated BMM from *Atg7^fl/fl^* or *Atg7^fl/fl^;Lyz2*-Cre mice were determined by ELISA. Data are expressed as means ± SDs. ****P* < .005, ##*P* < .01, and ###*P* < .005, One-way ANOVA.


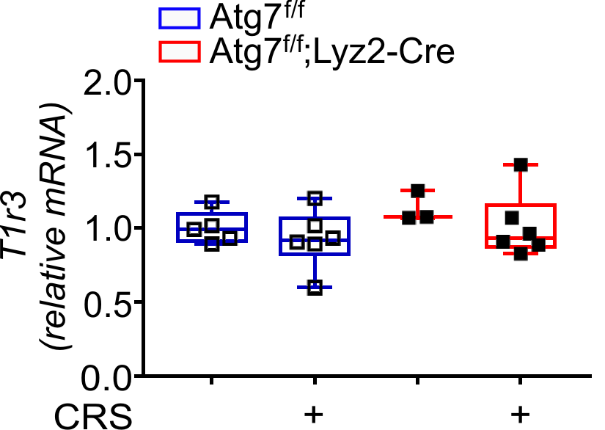


**Figure S8.** Expression of T1R3 in the sinonasal tissue of CRS mice.

Relative mRNA levels of T1R3 in the sinonasal tissue of normal or CRS mice were determined by using qRT-PCR with normalization to β-actin mRNA. Data are expressed as box-and-whisker plot with the box marking 25th, median, and 75th percentiles (*n* = 3-5 per group).


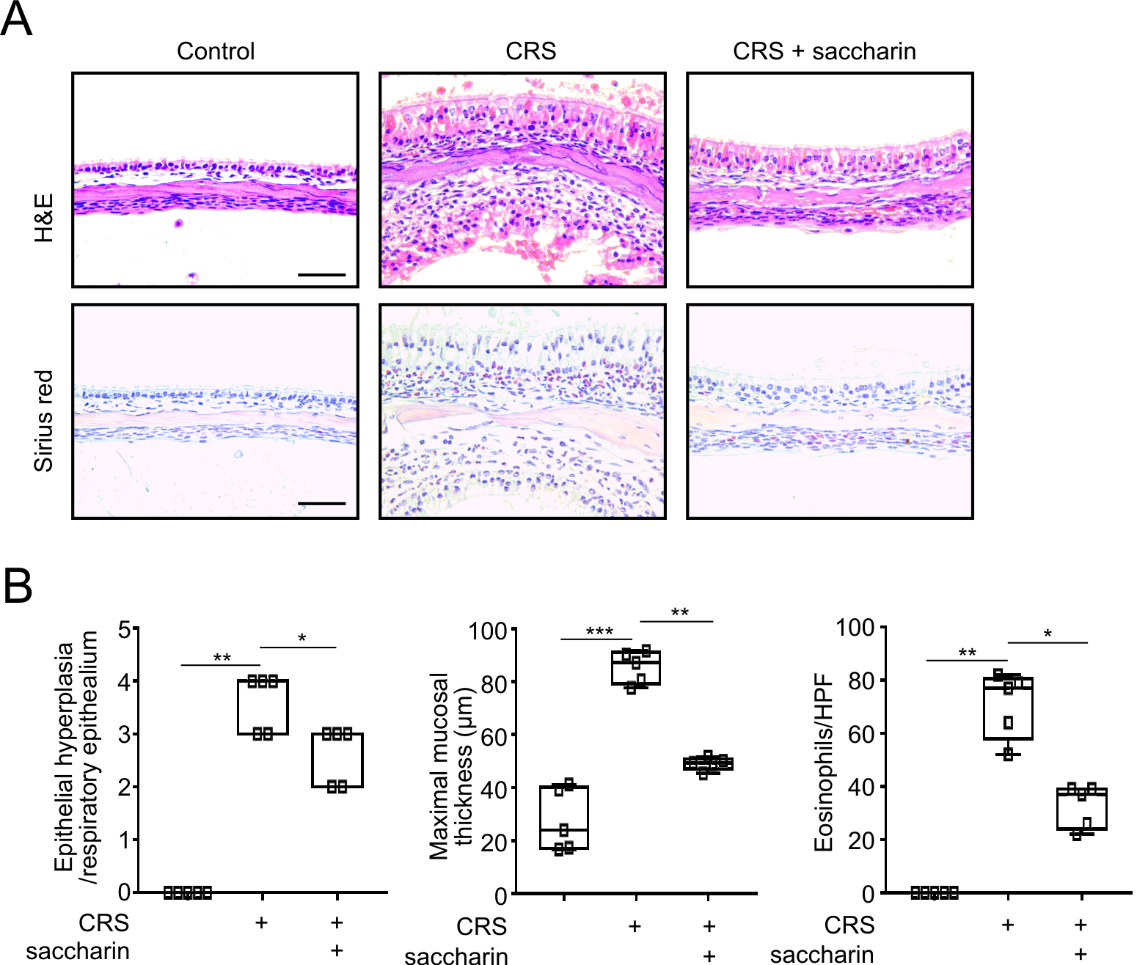


**Figure S9.** Saccharin alleviates eosinophilic inflammation in wild-type mice with ECRS.

(A) Effect of saccharin via intranasal (i.n.) injection on murine model of ECRS. Representative photographs of hematoxylin and eosin (H&E; *upper*)- and Sirius red (*lower*)-stained tissue sections. (B) Scores of epithelial hyperplasia, maximal mucosal thickness in H&E-stained tissue sections, and Sirius red-positive eosinophil counts of the lamina propria in Sirius red-stained tissue sections. Scale bars = 50 μm. Data are expressed as box-and-whisker plot with the box marking 25th, median, and 75th percentiles (*n* = 5 per group). **P* < .05, ***P* < .01, and ****P* < .005, Mann-Whitney *U* test.


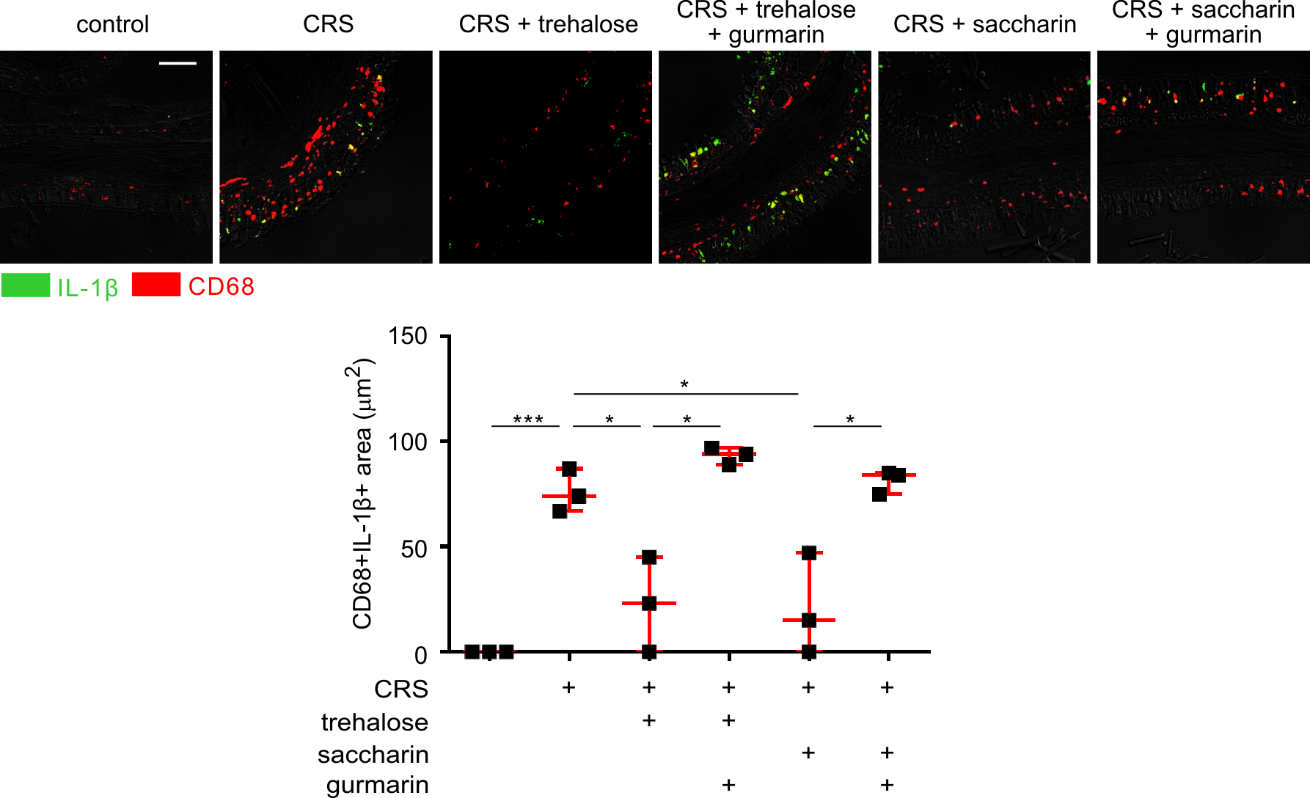


**Figure S10.** T1R3 blockade by gurmarin nullifies the effect of trehalose or saccharin on macrophage IL-1β regulation. Representative dual-immunofluorescence staining for IL-1β and CD68-positive macrophages in sinonasal tissue from each group of *Atg7^fl/fl^;Lyz2*-Cre mice (*upper*). Yellow signals indicate colocalization of 2 marker proteins. The statistical bar chart shows the area of colocalization (*lower*). Scale bars = 50 μm. Data are expressed as box-and-whisker plot with the box marking 25th, median, and 75th percentiles (*n* = 3 per group). **P* < .05, ****P* < .005, Mann-Whitney *U* test.


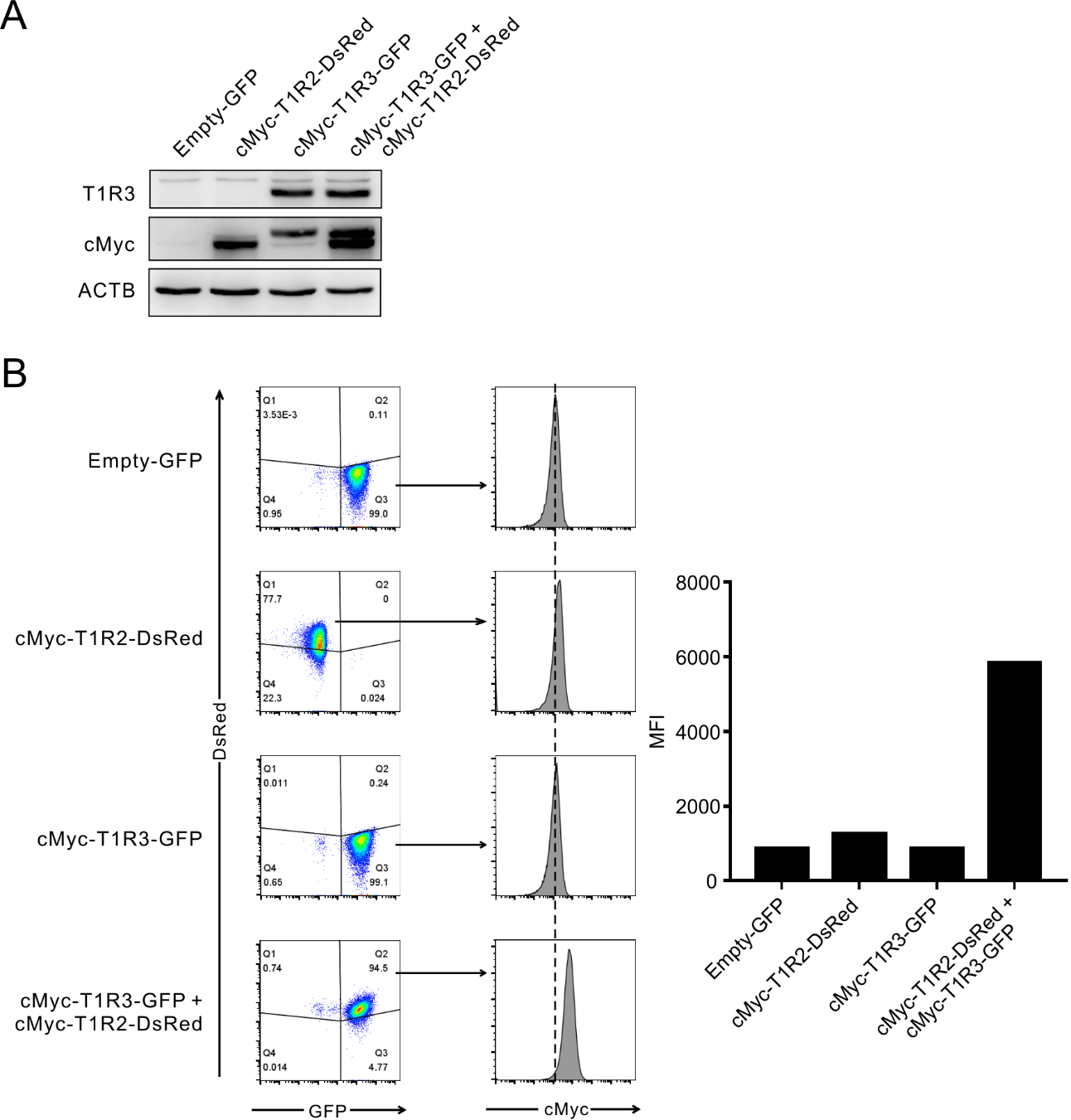


**Figure S11.** Overexpression of T1R3 in combination with T1R2 in THP-1 cells.

(A) Representative immunoblot analysis for T1R3, cMyc tag, and β-actin in lysates from THP-1 cells overexpressing human T1R3 and T1R2 alone or their combination. (B) Surface expression of T1R3 on THP-1 cells, as determined by staining with anti-cMyc antibody (*left*) and quantification by MFI value (*right*). Data are representative of three independent experiments.
